# Transcriptome and proteome profiling revealed molecular mechanism of selenium responses in bread wheat (*Triticum aestivum* L.)

**DOI:** 10.1186/s12870-021-03368-w

**Published:** 2021-12-09

**Authors:** Xiaoqing Feng, Qian Ma

**Affiliations:** Shandong Province Key Laboratory of Applied Mycology, College of Life Science, Qingdao Agricultural University, Qingdao, 266109 China

**Keywords:** Selenium, Bread wheat, Proteome, *Triticum aestivum*, Transcriptome

## Abstract

**Background:**

Although selenium (Se) plays important roles in scavenging free radicals, alleviating oxidative stresses, and strengthening immune system, the knowledge about Se responses in bread wheat is still limited. In order to clarify the molecular mechanism of Se responses in bread wheat, 2-week-old wheat seedlings of cultivar ‘Jimai22’ treated with 10 μM disodium selenate (Na_2_SeO_4_) for 0, 3, and 24 h were collected and analyzed by transcriptional sequencing and tandem mass tag-based (TMT) quantitative proteomics.

**Results:**

At least 11,656 proteins and 133,911 genes were identified, and proteins including ATP sulfurylase (APS), cysteine synthase (CS), SeCys lyase, sulfate transporters, glutathione S-transferase (GSTs), glutathione peroxidase (GSH-Px), glutaredoxins (GRXs), superoxide dismutases (SODs), catalases (CATs), heat shock proteins (HSPs), UDP-glycose flavonoid glycosyltransferases (UFGTs), sucrose-6-phosphate hydrolases (Suc-6-PHs), archaeal phosphoglucose isomerases (APGIs), malate synthases (MSs), and endo-1,4-beta-xylanase (Xyn) in Se accumulation, ROS scavenging, secondary metabolism, and carbohydrate metabolism were significantly differently expressed.

**Conclusions:**

This is the first complementary analyses of the transcriptome and proteome related with selenium responses in bread wheat. Our work enhances the understanding about the molecular mechanism of selenium responses in bread wheat.

**Supplementary Information:**

The online version contains supplementary material available at 10.1186/s12870-021-03368-w.

## Background

Selenium (Se) is a prosthetic group of many enzymes including glutathione peroxidase (GSH-Px), thioredoxin reductase (TrxR), and iodothyronine-deiodinase, so it plays important roles in scavenging free radicals, alleviating oxidative stresses, and strengthening immune systems [1]. Deficiency of Se in human diet causes growth retardation, bone metabolism impairment, and thyroid function abnormality, and increases the risk of Keshan disease, Kashin-Beck disease, muscle syndrome, liver disease, and many cancers [2]. Supplement of Se into the human dietary is one of most useful and common methods to solve Se deficient [3].

In plants, Se may be absorbed in the form of selenate (SeO_4_^2−^), selenite (SeO_3_^2−^), and organic Se. Selenate is the main form of Se in alkaline soils, whereas selenite is more predominantly presented in acid and neutral soils than other forms of Se [4]. Due to similar size and charge between selenate and sulfate (SO_4_^2−^), the selenate is taken in plants through sulfate transporters and then reduced to selenite [5].

The selenite is taken in plants by phosphate (PO_4_^3−^) transporters in the form of anions by a metabolically-dependent active process, although Se and phosphorus (P) are not in the same periodic group [1]. It was reported that increasing P supply significantly decreased the transportation and accumulation of Se in the winter wheat [6]. The selenite is reduced to selenide, and then incorporated into selenocysteine (SeCys), which can be converted into selenomethionine (SeMet) in plants [7]. It was reported that the selenite was rapidly assimilated into organic forms, and only small portion of inorganic Se were detected in wheat roots [8]. The SeCys, selenocystathionine, selenohomoserine, and SeMet are then assembled into selenoproteins. It was reported that SeMet was the major Se species in wheat grain samples [9], and SeMet and MeSeCys were the most abundant forms in Se-enriched plants [10].

The final content of Se in plants is controlled by uptake of Se from soil, assimilation of Se, and translocation of Se complexes into different organs. Se in low doses in plants was reported to protect the plants from variety of abiotic stresses including cold, drought, desiccation and heavy metals poisoning by decreasing reactive oxygen species (ROS) concentration, reducing electrolytic leakage and improving cell integrity [11–13]. Se has also been reported to delay senescence, increase crop production, improve nutritive value, increase respiratory potential, and protect the plants from pathogens, insects and herbivores [14, 15]. However, when Se content in plants exceeds the optimum concentration, Se toxicity occurs by malformed selenoproteins or induced oxidative stress [16]. Together, it was concluded that Se accumulation in plants should be kept in limited borders.

In the world, between 0.5 and 1 billion people have insufficient Se intake [17]. Supplement of Se into human dietary is one of the most useful and common methods to solve Se deficient [3]. However, low Se concentration in soil was detected in many regions in the world, including Western Europe, North Africa, and some parts of China [18]. Se fertilization is one of feasible strategies to enhance Se intake [19]. The other method to improve Se supplement is obtaining high Se accumulating crops by genetic engineering focused on manipulation of Se-related enzymes. Such as, overexpression of *Arabidopsis thaliana ATP sulfurylase* (*APS*) in *Brassica juncea* showed significantly improved Se accumulation [20, 21]. Together, understanding the molecular mechanism of Se responses in plants is meaningful.

Bread wheat (*Triticum aestivum* L., genome AABBDD) is one of the principal cereal grains produced and consumed globally. It is an allohexaploid that originated from hybridization between cultivated tetraploid wheat (*Triticum turgidum* L., BBAA) and the diploid wheat relative *Aegilops tauschii* Coss. (DD). A previous study has indicated that the diploid *Aegilops tauschii* has higher Se accumulation than the bread wheat [22]. Although bread wheat is more efficient in Se accumulation than other common cereal crops including rice, maize, barley and oats, it only recovered 20 ~ 35% of the applied Se fertilizer, indicating a low Se utilization efficiency [23]. Moreover, long term usage of Se fertilizers could be toxic to nearby ecosystem [24]. Hence, research on Se responses in bread wheat is meaningful to enhance human Se intake from dietary, and also important to keep plants in optimal Se concentration at the same time.

Two high-throughput transcriptional sequencing analysis has been used to uncover the molecular mechanism of Se responses in plants in recent years [3, 25]. In details, the transcriptional differences in both tender roots and young leaf tissues of tea plant with or without selenite treatment were analyzed by RNA-seq [25]. The young leaf of two genotypes of the diploid wheat relative *Aegilops tauschii* with contrasting Se-accumulating abilities with or without selenite treatments were also analyze by RNA-seq [3]. As we all known that most biological functions are carried out by proteins, detecting protein expression changes are more practical and valuable. Correlation analysis between proteomic and transcriptomic results provides information about transcriptional or post-transcriptional regulations of the related genes/proteins. However, there are still no reports about the Se responses by proteome analysis in plants.

In order to clarify the molecular mechanism in Se responses in bread wheat, 2-week-old wheat seedlings of cultivar ‘Jimai22’ treated with 10 μM disodium selenate (Na_2_SeO_4_) for 0, 3, and 12 h were collected and analyzed by transcriptional sequencing analysis and tandem mass tag-based (TMT) quantitative proteomics analysis in this research. The differentially expressed genes (DEGs) and proteins (DEPs) were analyzed by Gene Ontology (GO) enrichment analysis, Kyoto Encyclopedia of Genes and Genomes (KEGG) enrichment analysis, and correlation analysis, and the molecular mechanism in Se responses of bread wheat was also explored in this research.

## Results

### Different se accumulation in divergent wheat cultivars

In order to detect the differences of Se accumulation in divergent wheat cultivars, 5 main wheat cultivars in Huang and Huai River Wheat Zone of China including Jimai22 (JM22), Luyuan502 (LY502), Qingmai6 (QM6), WO4, and Jinan17 (JN17) were selected, and the Se contents in their dried seeds were detected. The results indicated that JM22 had highest Se contents in these 5 wheat cultivars, and the Se contents in JM22 was about 4 fold of others (Supplementary material 1). As a result, the wheat cultivar JM22 was chose for the following transcriptional analysis and proteomics analysis in this research.

### Gene identifications and DEGs analyses in transcriptomics

In order to clarify the molecular mechanism of Se responses in bread wheat, the 2-week-old wheat seedlings were treated with 10 μM Na_2_SeO_4_ for 0, 3, and 12 h, and the DEGs were detected by transcritomics in this research. The abbreviations of materials used in the transcriptomics were showed in Table 1. The results indicated that 133,911 genes were identified in each sample. Principal component analysis (PCA) showed the difference between groups was more significant than the variability among three replicates in a group, which indicated the reliability of data in this transcriptomic analysis (Supplementary material 2).

**Table 1:** The abbreviations of samples used in this research

Abbreviations	Detailed Information	Omics Used In
Se0hP	The seedlings of Na_2_SeO_3_ treated for 0 h	proteomics
Se3hP	The seedlings of Na_2_SeO_3_ treated for 3 h	proteomics
Se12hP	The seedlings of Na_2_SeO_3_ treated for 12 h	proteomics
Se0hR	The seedlings of Na_2_SeO_3_ treated for 0 h	transcriptomics
Se3hR	The seedlings of Na_2_SeO_3_ treated for 3 h	transcriptomics
Se12hR	The seedlings of Na_2_SeO_3_ treated for 12 h	transcriptomics

**Fig. 1:**
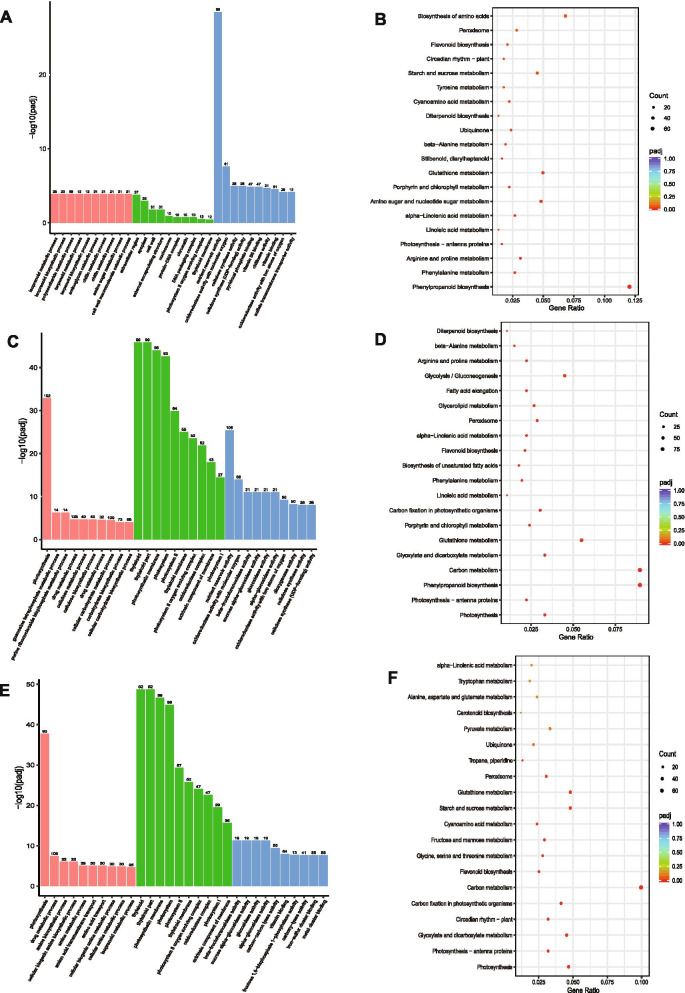
Comparisons between samples in different Se treatment periods in transcriptomic analysis. **A**, **C**, **E**, the GO enrichment analysis of DEGs between Se0hR and Se3hR samples (**A**), between Se0hR and Se12hR samples (**C**), and between Se3hR and Se12hR samples (**E**), respectively. The Y-axis indicated the significance level of the GO Term enrichment, represented by -log10(Padj), and the X-axis showed the processes/components in different biological processes (in red color), cellular components (in green color), and molecular functions (in blue color). **B**, **D**, **F**, the top 20 KEGG enriched scatter plot of DEGs between Se0hR and Se3hR samples (**B**), between Se0hR and Se12hR samples (**D**), and between Se3hR and Se12hR samples (**F**), respectively. The X-axis referred to the ratio of the gene number enriched in the pathway to the number of annotated genes. The bigger the Rich factor, the more significant the enrichment was. The Y-axis was the corrected *p*-value after multiple hypotheses testing, which was ranged from 0 to 1. The closer to zero, the more significant the enrichment was

The genes with corrected P-value < 0.05 and absolute fold change ≥2 were considered as significant DEGs. In order to detect the genes in Se responses of bread wheat, the significant DEGs between two samples of Se0hR, Se3hR and Se12hR were detected. The results showed that 8258 DEGs were differentially expressed between Se0hR and Se3hR samples, which included 4806 up-regulated DEGs and 3452 down-regulated DEGs (Supplementary material 3). Analyzing these DEGs by GO and KEGG enrichment analyses indicated that the DEGs after Se treatment for 3 h including *UDP-glycose flavonoid glycosyltransferase*s (*UFGT*s), *cytochrome P450*s (*CYP*s), *GDSL lipase*s, and *sucrose-6-phosphate hydrolase*s (*Suc-6-PH*s) were mainly in peroxisome, flavonoid biosynthesis, starch and sucrose metabolism, and glutathione metabolism (Fig. 1, Supplementary material 3).

There were 14,633 DEGs between Se0hR and Se12hR samples, which included up-regulated 6694 DEGs and 7939 down-regulated DEGs (Supplementary material 3). Analyzing these DEGs by GO and KEGG enrichment analyses indicated that the DEGs after Se treatment for 12 h including *CYP*s, *UFGT*s, *carboxypeptidase C*s (*CPC*s), *Suc-6-PH*s, and *ubiquinone*s (*UQ*s) were mainly in diterpenoid biosynthesis, glycolysis, fatty acid elongation, glycerolipid metabolism, and peroxisome (Fig. 1, Supplementary material 3).

There were 9743 DEGs between Se3hR and Se12hR samples, which included 3630 up-regulated and 6113 down-regulated (Supplementary material 3). Analyzing these DEGs by GO and KEGG enrichment analyses indicated that the DEGs in the comparison between Se3hR and Se12hR samples including *CYP*s, *UFGT*s, *CPC*s, *catalase*s *(CAT*s), and *liposygenase*s (*LOX*s) were mainly in carbon metabolism, photosynthesis, starch and sucrose metabolism, and glutathione metabolism (Fig. 1, Supplementary material 3).

### Protein identifications and DEPs analyses in proteomics

In order to clarify the molecular mechanism of Se responses in bread wheat, the 2-week-old wheat seedlings were treated with 10 μM Na_2_SeO_4_ for 0, 3, and 12 h, and the expressed proteins were detected by TMT proteomics in this research. The abbreviations of materials used in the proteomics were showed in Table 1. The results indicated that 11,656 proteins were identified. PCA analysis showed the difference between groups was more significant than the variability among three replicates in a group, which indicated the reliability of data in this proteomic analysis (Supplementary material 4).

Proteins with fold change in a comparison > 1.2 or < 0.83 and unadjusted significance level *p* < 0.05 were considered as DEPs. In order to detect the proteins in Se responses, the significant DEPs between Se0hP and Se3hP samples, between Se0hP and Se12hP samples, and between Se3hP and Se12hP samples were detected.

### DEPs analysis between Se0hP and Se3hP samples

In order to clarify DEPs after Se treatment for 3 h in this research, a single comparison between Se0hP and Se3hP samples was conducted, and the results indicated that 273 DEPs including 130 up-regulated DEPs and 143 down-regulated DEPs were differentially expressed due to Se treatment for 3 h, (Supplementary material 5).

**Fig. 2:**
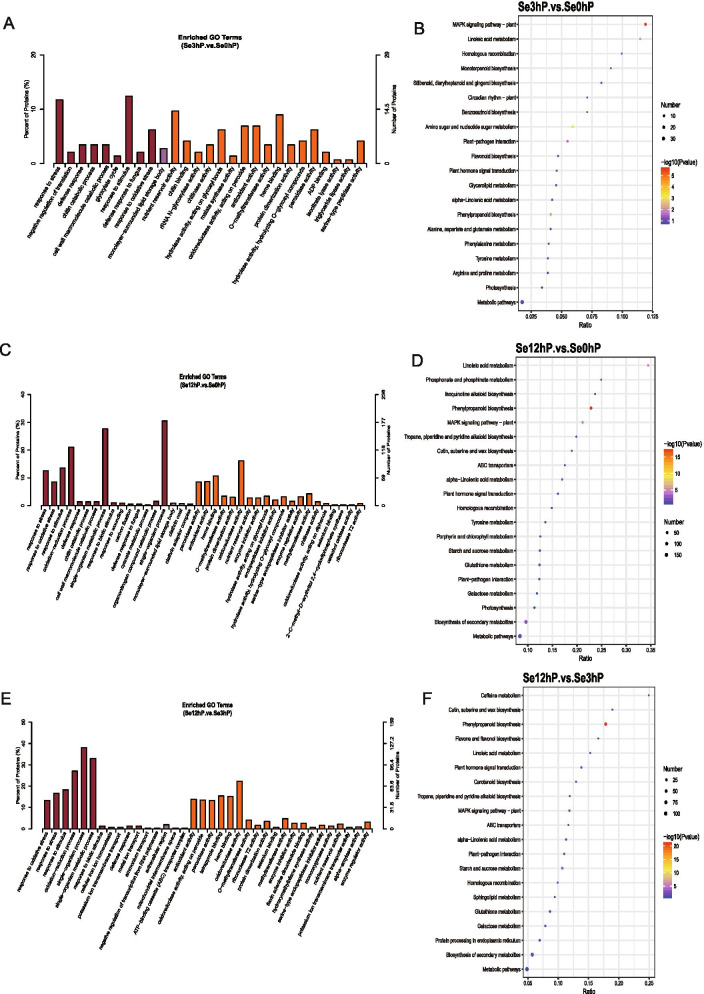
Comparisons between samples in different Se treatment periods in proteomic analysis. **A**, **C**, **E**, the GO enrichment analysis of DEPs between Se0hP and Se3hP samples (**A**), between Se0hP and Se12hP samples (**C**), and between Se3hP and Se12hP samples (**E**), respectively. The Y-axis indicated the number of DEPs or percentage of proteins, and the X-axis showed the processes/components in different biological processes (in red color), cellular components (in purple color), and molecular functions (in orange color). **B**, **D**, **F**, the top 20 KEGG enriched scatter plot of DEPs between Se0hP and Se3hP samples (**B**), between Se0hP and Se12hP samples (**D**), and between Se3hP and Se12hP samples (**F**), respectively. The X-axis referred to the ratio of the protein number enriched in the pathway to the number of annotated proteins. The bigger the Rich factor, the more significant the enrichment was. The Y-axis was the corrected *p*-value after multiple hypotheses testing, which was ranged from 0 to 1. The closer to zero, the more significant the enrichment was

Analyzing these 273 DEPs by GO and KEGG enrichment analyses indicated that the DEPs after Se treatment for 3 h were mainly in plant hormone signaling, photosynthesis, monoterpenoid and flavonoid biosynthesis, and phenylalanine, tyrosine, arginine, proline, and glycerolipid metabolism, which were related with stress response, stimulus response, nutrient reservoir activity, antioxidant activity, hydrolase activity, and peroxidase activity (Fig. 2, Supplementary material 5). Analyzing the DEPs in these processes showed that the expression of CPCs, malate synthases (MSs), heat shock proteins (HSPs), Leucine-rich repeat proteins (LRRs), CYPs, Suc-6-PHs, UFGTs, and endo-1,4-beta-xylanase (Xyn) were significantly changed (Supplementary material 5).

### DEPs analysis between Se0hP and Se12hP samples

In order to clarify DEPs after Se treatment for 12 h, a single comparison between Se0hP and Se12hP samples was also conducted, and the results indicated that 952 DEPs including 381 up-regulated DEPs and 571 down-regulated DEPs were differentially expressed after 12 h Se treatment (Supplementary material 5).

Analyzing these 952 DEPs by GO and KEGG enrichment analyses indicated that the DEPs after Se treatment for 12 h were mainly in linoleic acid metabolism, phosphonate and phosphinate metabolism, cutin, suberine and wax biosynthesis, phenylpropanoid biosynthesis, and plant hormone signaling, which were related with stress stimulus, oxidoreductase activity, antioxidant activity, and peroxidase activity (Fig. 2, Supplementary material 5). Analyzing the DEPs in these processes showed that the expression of UQs, chitinases, pathogenesis-related protein1s (PR1s), UFGTs, Suc-6-PHs, HSPs, glycogen synthases (GSs), and CATs were significantly changed (Supplementary material 5).

### DEPs analysis between Se3hP and Se12hP samples

In order to clarify DEPs in different Se treatment periods, the Se3hP and Se12hP samples were compared, and the results indicated that 554 DEPs including 226 up-regulated and 328 down-regulated were differentially expressed between Se treatment 3 and 12 h (Supplementary material 5).

Analyzing these 554 DEPs by GO and KEGG enrichment analyses indicated that the DEPs between Se3hP and Se12hP samples focused on caffeine metabolism, cutin, suberine and wax biosynthesis, phenylpropanoid biosynthesis, and flavone and flavonol biosynthesis, which related with oxidoreductase activity, peroxidase activity and antioxidant activity (Fig. 2, Supplementary material 5). Analyzing the DEPs in these processes showed that the expression of UQs, PR1s, LRRs, glutathione S-transferases (GSTs), K^+^ transporters, CATs, HSPs, CYPs, and CPCs were significantly changed (Supplementary material 5).

### Correlation between the proteomic and transcriptomic results

**Fig. 3:**
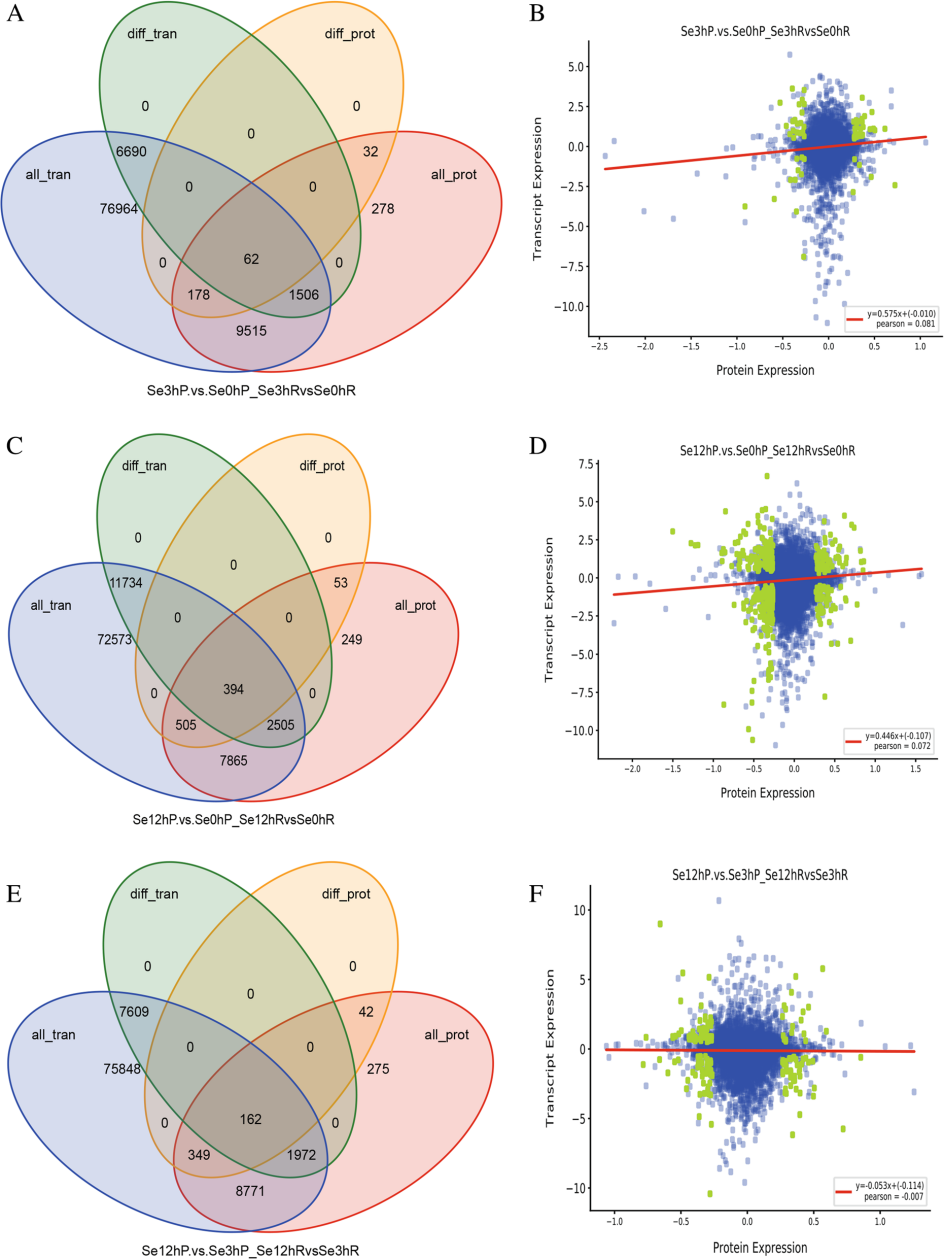
Correlation analysis between proteome and transcriptome by Venn diagrams and scatter plot of expression correlation. **A**, **C**, **E**, the Venn diagrams of genes, proteins, DEGs, and DEPs between Se0h and Se3h samples (**A**), between Se0h and Se12h samples (**C**), and between Se3h and Se12h samples (**E**), respectively. all_ tran represented all the genes obtained from the transcriptome, diff_ tran represented the DEGs identified by transcriptome, all_ prot represented all the proteins identified by proteome, and diff_ prot represented the DEPs identified by proteome. **B**, **D**, **F**, the scatter plot of expression correlation between Se0hP and Se3hP samples (**B**), between Se0hP and Se12hP samples (**D**), and between Se3hP and Se12hP samples (**F**), respectively. The abscissa was the differential multiple of proteins, the ordinate was the differential multiple of corresponding genes, and the correlation coefficient and *P* value of transcriptome and proteome were also showed in the figures. Each point represented a protein, the blue point represented non-differential proteins, and the green point represented DEPs

Analyzing the results of transcriptome and proteome indicated that there were 62 and 394 genes/proteins differentially expressed in both gene and protein levels after Se treatment for 3 and 12 h, respectively (Fig. 3, Supplementary material 6). These genes probably functioned in the Se response pathways in the bread wheat. And 162 genes/proteins were differentially expressed in both gene and protein levels between se treatment for 3 and 12 h, indicating these 162 genes/proteins played roles in Se response pathways, but in different Se treatment periods (Fig. 3, Supplementary material 6). It was reported that most of genes are divided into early response genes that are activated at the transcription level in the first round of response to stimuli before any new proteins are synthesized and late response genes that are induced following the synthesis of early response gene products [26, 27]. These DEPs/DEGs probably belonged to the early response genes.

The results also indicated that 1506, 2505 and 1972 genes were detected differentially expressed in gene level but not in protein level in the comparisons between Se0h and Se3h samples, between Se0h and Se12h samples, and between Se3h and Se12h samples, respectively (Fig. 3, Supplementary material 6). The expression pattern of these genes hinted that these genes probably belonged to the late response genes in Se response pathways. Because of the limited treatment and sampling time in this research, these proteins had not been translated, and the changes of their proteins level had not been detected.

The correlation analysis between proteome and transcriptome was also indicated that 178, 505, and 349 proteins were differentially expressed in protein level but not in gene level in the comparisons between Se0h and Se3h samples, between Se0h and Se12h samples, and between Se3h and Se12h samples, respectively (Fig. 3, Supplementary material 6). Considering these DEPs only detected in protein level but not in gene level, it was concluded that the modification and activation in post-transcription level were might present in these proteins, which released active proteins quickly without transcription of the corresponding genes.

### Verification of RNA-seq results by quantitative real time PCR (qRT-PCR)

**Fig. 4:**
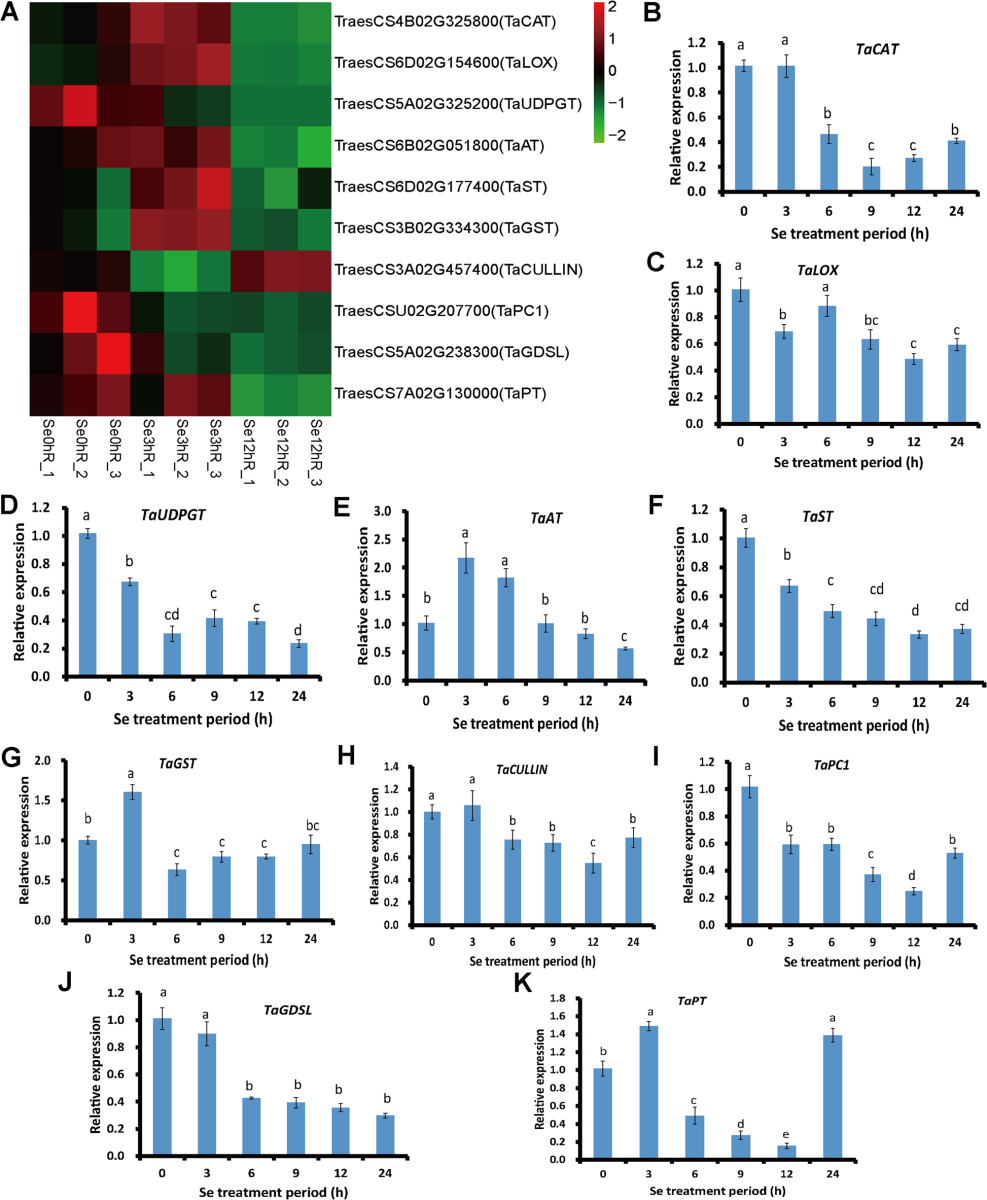
Relative expression of 10 randomly selected DEGs in this research. A, heatmap of relative expression level of the selected DEGs by RNA-seq. B-K, the relative expression of the selected DEGs by qRT-PCR. The statistical significance of the difference was confirmed by ANOVA at α = 0.05 level

In order to confirm the results of RNA-seq and detect the roles of functional genes in Se responses in the bread wheat, 10 DEGs were randomly selected out of 133,911 identified genes, and their relative expression levels in Se treatment for 0, 3, 6, 9, 12, and 24 h were estimated by qRT-PCR (Fig. 4, Supplementary material 3). Their relative expression levels in qRT-PCR were compared with the results of RNA-seq, and the results showed high consistent in these two methods, which indicated that the sequencing results were dependable. Because the detected 10 DEGs in qRT-PCR had already been proved playing roles in Se responses of bread wheat by RNA-seq, the selection of assessed DEGs could be biased.

### Comparisons of se accumulation related genes in different se content cultivars

**Fig. 5:**
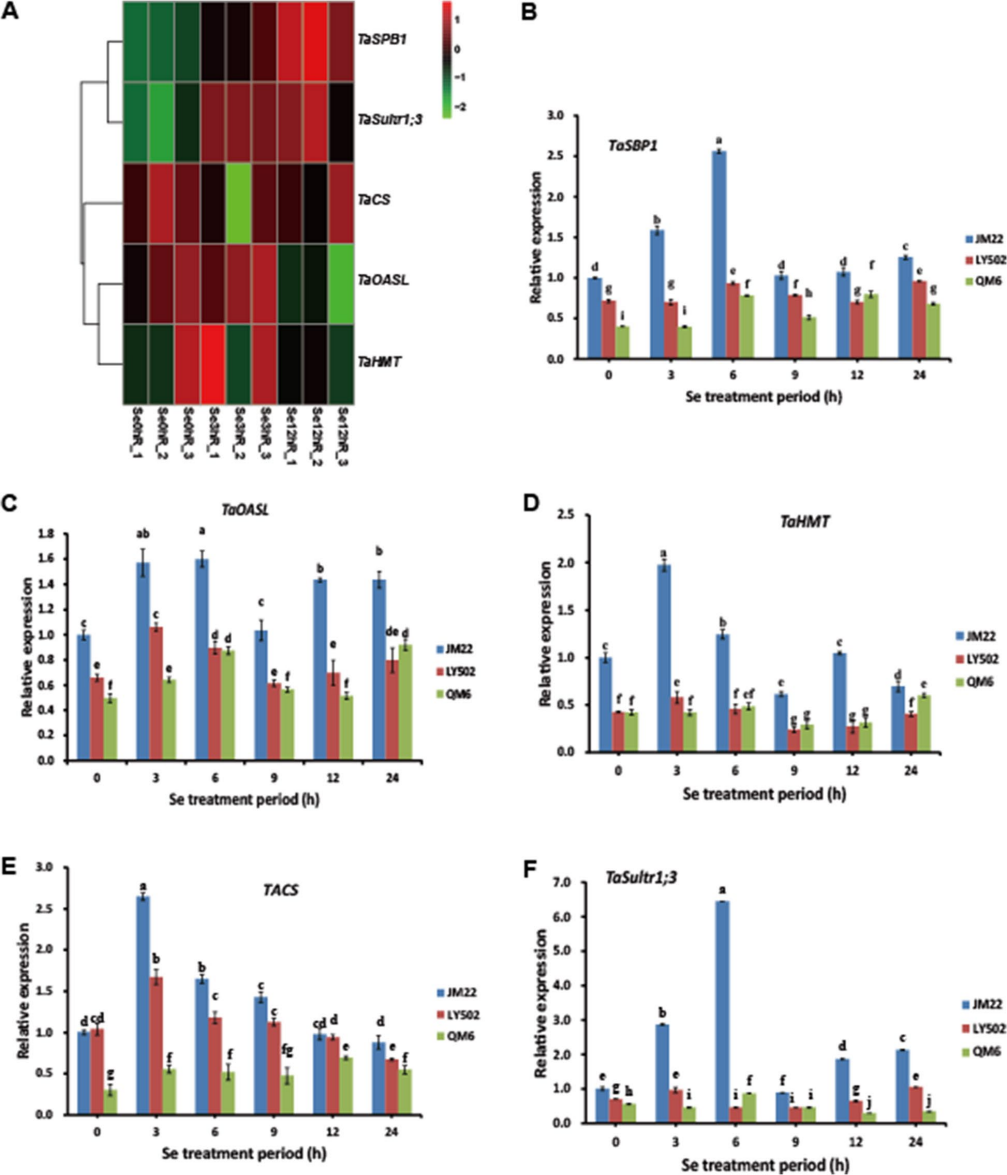
Relative expression of Se accumulation related genes in different wheat cultivars. **A**, heatmap of relative expression level of Se accumulation related genes by RNA-seq. **B**-**F**, the relative expression of Se accumulation related genes by qRT-PCR. The statistical significance of the difference was confirmed by ANOVA at α = 0.05 level

In order to detect the relationship between expression level of Se accumulation related genes and Se contents in wheat seeds, 5 Se uptake and transportation related genes including *TaSBP1*, *TaOASL*, *TaHMT*, *TaCS* and *TaSultr1;3* were selected, and their expression levels in this transcriptional analysis were detected. The results indicated that the expression of *TaSPB1* and *TaSultr1;3* was activated and increased after Se treatment (Fig. 5A). The expression of *TaCS* decreased firstly and then began to increase, while the expression of *TaOASL* and *TaHMT* increased to the peak at 3 h of Se treatment and then began to decrease (Fig. 5A). Together, the results of transcriptional analysis confirmed their functions in Se accumulation in wheat cultivar JM22 with high seeds Se contents.

In order to explore their functions in wheat cultivars with low seeds Se contents, the expression of Se accumulation related genes *TaSPB1*, *TaSultr1;3*, *TaCS*, *TaOASL* and *TaHMT* in 3 wheat cultivars with divergent seeds Se contents was detected by qRT-PCR. The results indicated that the expression of *TaHMT* and *TaCS* increased due to Se treatment with a peak of transcript accumulation observed after 3 h of treatment, while the expression of *TaSBP1*, *TaOASL*, and *TaSultr1;3* arrived the peak after 6 h of treatment in the JM22 with high seeds Se contents (Fig. 5B-F). The expression pattern of these 5 genes in LY502 and QM6 were similar with JM22, but the expression levels of these 5 Se accumulation related genes in LY502 and QM6 with low seeds Se contents were lower than their expression levels in JM22 with high Se contents in seeds (Fig. 5B-F), which probably rendered to their different Se accumulation in seeds (Supplementary material 1).

### Physiological index changes due to se treatment in bread wheat

**Fig. 6:**
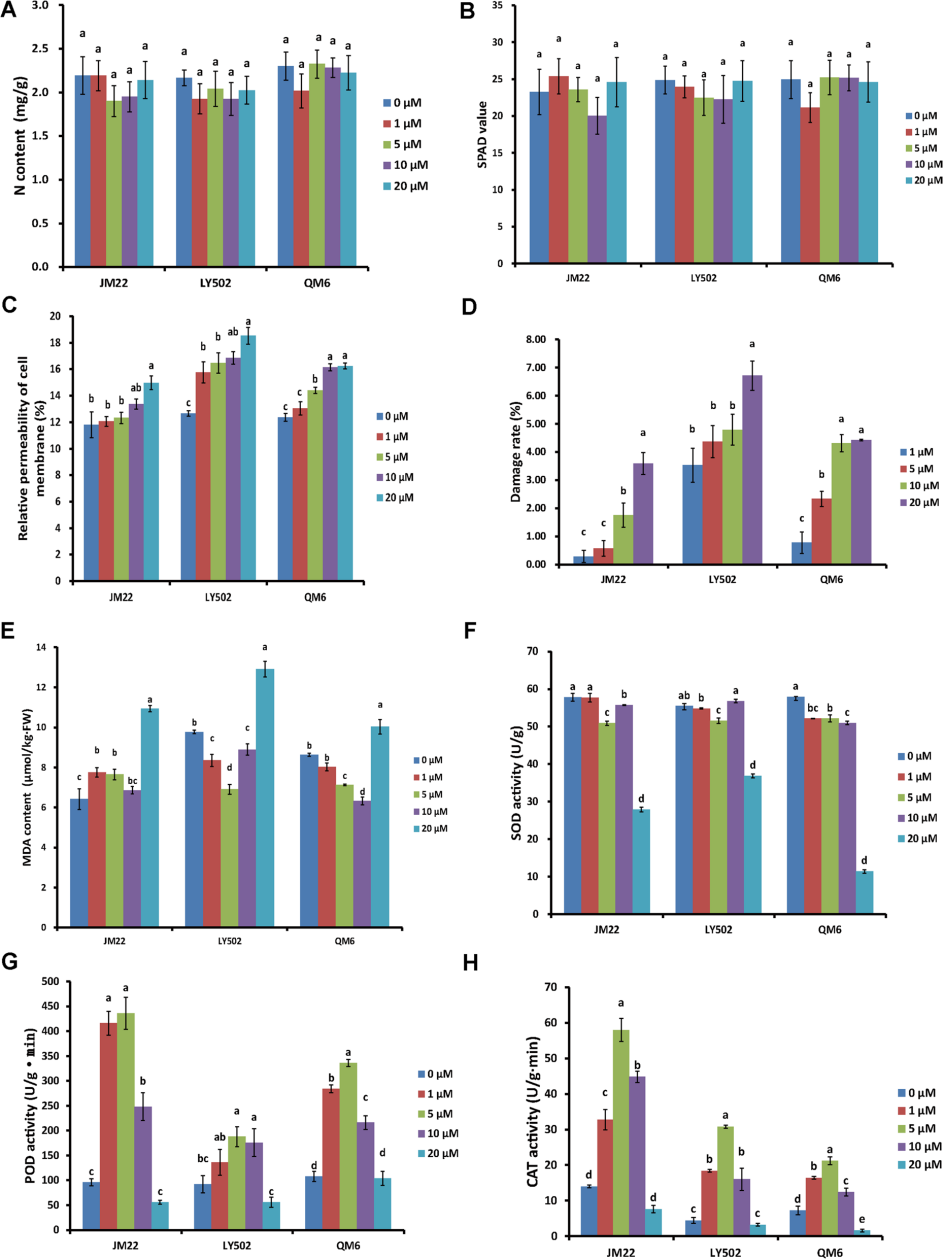
Detection of physiological indexes of bread wheat seedlings with different Se concentration treatments. The nitrogen content (**A**), relative content of total chlorophyll (**B**), relative permeability of cell membrane (**C**), damage rate of leaves (**D**), MDA content (**E**), SOD activity (**F**), POD activity (**G**), and CAT activity (H) of bread wheat seedlings were detected, and the statistical significance of the difference was confirmed by ANOVA at α = 0.05 level

The DEPs/DEGs related with the stress stimulus, oxidoreductase activity, and antioxidant activity, were detected in the Se responses of bread wheat in this research (Fig. 2, Supplementary material 3,5,6). In order to confirm the results of transcriptional and proteomic analyses, the nitrogen content, relative content of total chlorophyll, relative permeability of cell membrane, damage rate of leaves, malondialdehyde (MDA) content, superoxide dismutase (SOD) activity, peroxidase (POD) activity, and CAT activity were detected, and the results indicated that the Na_2_SeO_4_ treatment had no significant effects on nitrogen content and relative content of total chlorophyll of bread wheat seedlings (Fig. 6A-B). The relative permeability of cell membrane and damage rate of leaves were enhanced as the increasing of Na_2_SeO_4_ concentration (Fig. 6C-D). The SOD, POD and CAT activities were enhanced at low concentration of Na_2_SeO_4_ treatment, but their activities were decreased significantly when the Na_2_SeO_4_ concentration was increased to 20 μM, and as a result, the MDA contents that are produced by membrane lipid peroxidation and reflect the degree of plant suffering from stresses, were increased dramatically at 20 μM Na_2_SeO_4_ treatment (Fig. 6E-H). Together, it was concluded that the Se treatment induced significant changes in antioxidant related proteins as the results detected in transcriptional and proteomic analyses. These results also indicated that low concentration of Se treatment improved antioxidant activities and made the bread wheat seedlings stronger, but high concentration of Se treatment was harmful to the growth of bread wheat seedlings, which was consistent with previous reports [13, 16].

## Discussion

Se is important for human health, deficiency of Se in dietary causes a serious of diseases, and between 0.5 and 1 billion people have insufficient Se intake in the world [17]. Supplement of Se into the human dietary is one of most useful and common methods to solve Se deficient [3]. Se is also important for plants. It was reported that Se in low doses protects plants from abiotic and biotic stresses, while high concentration of Se in plants induces oxidative stresses on the contrary [16]. Bread wheat is one of the principal cereal grains produced and consumed globally, and the Se also affects bread wheat growth, development and biotic and abiotic resistance, so the Se responses in bread wheat were detected by transcriptional and proteomic analyses in this research. The molecular mechanism of Se responses in bread wheat was uncovered.

### Proteins related with se accumulation were accelerated after se treatment

In plants, the selenate is absorbed through sulfate transporters and the selenite is taken by phosphate transporters in an active process [1]. The absorbed selenate is converted to selenite by two enzymes APS and APS reductase (APR). The APS catalyzes the hydrolysis of ATP to form adenosine phosphoselenate, which is then reduced to selenite by APR [28]. The selenite is then converted to selenide by glutathione or glutaredoxins (GRXs) in plants [29]. Selenide is converted to SeCys by enzyme cysteine synthase (CS). SeCys is then converted to elemental Se by Cys lyase, or is methylated to methyl-SeCys (Me-SeCys) by selenocysteine methyltransferase, or is converted to SeMet by a series of enzymes in different plant species and different environmental conditions.

In this research, the proteins related with Se accumulation were detected significantly changed in the bread wheat seedlings (Supplementary material 3,5,6). In details, 13 sulfate transporters were detected in Se transportation. The expressions of sulfate transporter coding genes TraesCS7A02G088700, TraesCS4A02G029100, and TraesCS4B02G264100 were increased sharply in first 3 h, and then decreased quickly. As a result, their expression changes were not significant any more after 12 h of Se treatment. The expression of TraesCS4D02G264200 was increased sharply in first 3 h, and then decreased quickly. However, its expression changes were still significant after 12 h of Se treatment. These results indicated these 4 sulfate transporter coding genes probably function in the early hours of Se treatment. However, the expression of sulfate transporters coding genes TraesCS3A02G288800, TraesCS4A02G043400, TraesCS4B02G263900, TraesCS4D02G264100, TraesCS4A02G043500, TraesCS5D02G237800, TraesCS5A02G229700, TraesCS5B02G163700, and TraesCS2A02G508200 were increased slowly in first 3 h, and their expression changes were finally detected significantly changed after 12 h of continuing increase, indicating these 9 sulfate transporter coding genes playing important roles in late hours of Se treatment.

The APS coding gene TraesCS2A02G032500 were changed significantly after 12 h of Se treatment in both protein and RNA level (Supplementary material 3,5,6), which meant the assimilation of selenite was significantly improved in 12 h after Se treatment and the APS was important in Se accumulation of bread wheat. This result was consistent with previous reports. Such as, the *APS* genes have been detected by Se treatment in *Astragalus chrysochlorus* by RNA-Seq [30]. Overexpression of *Arabidopsis thaliana AtAP*S in *Brassica juncea* resulted in significantly improved Se accumulation [20, 21]. It was reported that the expression of *CS* is related with Se accumulation in leaves of plants [25, 31, 32]. The CS enzyme coding gene novel.8735 was also significantly changed only in RNA level after 12 h of Se treatment in bread wheat in this research (Supplementary material 3-5). The SeCys lyase TraesCS5B02G407300 was significantly changed only in protein level after 12 h of Se treatment in this research (Supplementary material 3,5,6). These genes were also proved playing important roles in Se assimilation in this research.

### ROS scavenging enzymes functioned in se responses

It was reported that Se in low doses protects the plants from variety of abiotic stresses by decreasing ROS concentration [11–13]. GSTs protect cells from oxidative damages by combining excess toxin with glutathione and forming, transferring to and separating S-glutathione conjugates in the vacuole [33]. SODs catalyze the dismutation of superoxide radicals to produce hydrogen peroxide (H_2_O_2_), which is decomposed into oxygen and water by CAT in plants.

In this research, the protein expression levels of 20 GSTs (TraesCS2A02G578400, TraesCS2B02G244100, TraesCS3A02G437400, TraesCS3B02G539100, TraesCS3D02G486100, etc.), 1 GSH-Px (TraesCS2D02G407700), 1 GRX (TraesCS6B02G361200), 3 SODs (TraesCS2D02G538300, TraesCS7D02G290700, and TraesCS7A02G292100), and 3 CATs (TraesCS6B02G462300, TraesCS7B02G140600, and TraesCS5A02G113100) were significantly changed after Se treatment (Supplementary material 5). There are more genes related with ROS scavenging were detected in the transcriptional analysis in this research (Supplementary material 3). Transcriptional analysis of tea plant also showed that ROS scavenging related *GST*s, *glutathione synthetase*s (*GSS*s), *GSH-Px*, *glutathione reductase*s (*GR*s), *GRX*s, and *CAT*s were detected significantly changed after selenite treatment [25]. Antioxidant *GST*s and *CAT*s were proved in the Se response pathway in *Stanleya pinnata* and *Arabidopsis thaliana* [32, 34].

### Chaperons played roles in se responses

Chaperon proteins including HSPs improve protein stability by regulating protein folding, localization, accumulation and degradation under multiple abiotic stresses treatments, such as heat, cold, salt, oxidative, and heavy metals in plants [35, 36].

In this research, 15 chaperon proteins were detected differentially expressed in the Se response pathway, including 2 HSP90s (TraesCS7D02G241100 and TraesCS2B02G047400), 2 HSP70s (TraesCS1A02G133100 and TraesCS1A02G285000), 9 HSP20s (TraesCS1B02G471900, TraesCS3A02G113000, TraesCS4A02G092100, TraesCS4B02G212300, TraesCS4D02G212500, TraesCS6D02G322300, TraesCS6A02G181700, TraesCS2A02G312900, and TraesCS3A02G034500), and 2 other chaperon proteins (TraesCS2B02G320000 and TraesCS5D02G497200) (Supplementary material 5). In transcriptional analysis, 15 DEGs were identified including TraesCS4A02G098600, TraesCS5A02G268100, TraesCS1B02G151300, TraesCS2B02G374700, TraesCS5D02G492900, TraesCS5B02G492500, TraesCS5A02G479300, TraesCS5B02G267900, TraesCS1D02G284000, TraesCS1B02G294300, TraesCS6A02G342400, TraesCS4B02G142400, TraesCS6B02G058300, TraesCS4B02G206300, and TraesCS5A02G078000 (Supplementary material 3). Unexpectedly, there were no overlap between the proteomic analysis results and transcriptional analysis results. As a result, it was concluded that these 30 chaperon proteins probably functioned in Se response pathway.

### Secondary metabolisms were enhanced due to se treatment

Secondary metabolisms produce a serious of small compounds called secondary metabolites, which include basic nutrients such as proteins, fats or carbohydrates, and other compounds such as taxoids, polysaccharides, flavones. These secondary metabolites are dispensable for plant metabolism and growth, and tolerance to both biotic and abiotic stresses [37]. The transcriptional analysis of diploid wheat relative *Aegilops tauschii* (DD) after Se treatment indicated that DEGs involved in flavone and flavonol biosynthesis, flavonoid biosynthesis, and selenocompound metabolism were believed to be potentially related to selenium metabolism [3].

In this research, 14 UFGTs (TraesCS2B02G040500, TraesCS2B02G081400, TraesCS2D02G069100, TraesCS3D02G120200, TraesCS5B02G436300, TraesCS5D02G440900, TraesCS5D02G476400, TraesCS7A02G492800, TraesCS7B02G074700, TraesCS4B02G226700, TraesCS6D02G162700, TraesCSU02G009000, TraesCS1B02G062100, and TraesCS2A02G273800) were differentially expressed in protein level after Se treatment of bread wheat (Supplementary material 5). In transcriptional analysis, 124 and 192 *UFGTs* were differentially expressed after Se treatment of bread wheat (Supplementary material 3). UFGTs are the last enzyme in anthocyanin synthesis process, which catalyze unstable anthocyanins into stable anthocyanins, so the activity of UFGTs is positively correlated with anthocyanins synthesis. Together, it was concluded that anthocyanin synthesis was enhanced due to Se treatment.

### Carbohydrate metabolism was changed after se treatment

In this research, 10 Suc-6-PHs (TraesCS7D02G008800, TraesCS7D02G009400, TraesCS2A02G588300, TraesCS2B02G594900, TraesCS2D02G489200, TraesCS4A02G485600, TraesCS7A02G009200, TraesCS7A02G009800, TraesCS7D02G010000, and TraesCS2A02G488900), 4 archaeal phosphoglucose isomerases (APGIs) (TraesCS6D02G367000, TraesCS4D02G031900, TraesCS6D02G367700, and TraesCSU02G137500), 2 MSs including TraesCS2A02G345500 and TraesCS2D02G344200, and 1 Xyn (TraesCS5D02G448800) were differentially expressed in protein level (Supplementary material 5).

It was reported that Suc-6-PH hydrolyzes the terminal non-reducing beta-D-fructofuranoside residues in beta-D-fructofuranosides, which involves in sucrose metabolism and glycan biosynthesis. Phosphoglucose isomerases catalyze glucose 6- phosphate to form fructose 6-phosphate. The MSs combine glyoxylic acid with acetyl CoA to form malic acid in photosynthesis. The Xyn hydrolyzes 1,4-beta-D-xylosidic linkages in xylans, and is involved in the xylan degradation pathway and glycan degradation. In this research, 1 Xyn (TraesCS5D02G448800) decreased after Se treatment of bread wheat, indicating the Se treatment prevented the xylan degradation in bread wheat. Together, Se treatment affected the carbohydrate metabolism in the bread wheat.

## Conclusion

The proposed molecular mechanism of Se responses in bread wheat is started with increase of Se accumulation related proteins including APS, CS, SeCys lyase, and sulfate transporters. Then, ROS scavenging enzymes (GSTs, GSH-Px, GRXs, SODs, and CATs) and chaperons (HSP90s, HSP70s, and HSP20s) are induced, secondary metabolism (UFGTs) is enhanced, and carbohydrate metabolism (Suc-6-PHs, APGIs, MSs, and Xyn) is changed due to Se treatment. The genes/proteins in same family are expressed in different regulation mechanisms and play important roles in different stages. Of course, other proteins in unclear pathways are also initiated and probably play important roles.

## Methods

### Plant material treatment and sample collection

The bread wheat (*Triticum aestivum* L.) cultivar JM22 cultivated by Shandong Academy of Agriculture and Science (Jinan, Shandong Province, China) was used in this research. The JM22 was a popular wheat cultivar with high yield, multi-resistance, and high quality medium gluten, which derived from hybridization of ‘935024’ and ‘935106’ in Shandong Province of China in 2006. JM22 is one of main wheat cultivars in the north of Huang and Huai River Wheat Zone of China.

The seeds of JM22, LY502, QM6, WO4, and JN17 used in this research were harvested from the Se-rich soils of Yanzhou in Shandong Province, China. In this research, 2-week-old wheat seedlings of wheat cultivars were sprayed with 10 μM Na_2_SeO_4_ for 0, 3, 6, 9, 12, and 24 h, respectively [3]. The whole plants were collected and frozen in liquid nitrogen. The samples of JM22 treated with 10 μM Na_2_SeO_4_ for 0, 3, and 12 h were used for transcriptional analysis and proteomic analysis. The abbreviations of materials used in the transcriptome and proteome were showed in Table 1. The seedlings of JM22, LY502 and QM6 treated with 10 μM Na_2_SeO_4_ for 0, 3, 6, 9, 12, and 24 h were used for the qRT-PCR analysis.

### Transcriptome sequencing and data analysis

In this research, three independent biological replicates were used, and at least 10 whole seedlings were mixed in each replicates. A total amount of 1 μg RNA per sample was used for library preparation, and the library quality was assessed on the Agilent Bioanalyzer 2100 system. The library preparations were sequenced on an Illumina Novaseq platform by Novogene Bioinformatics Technology Co. Ltd. (Beijing). The raw data of FASTQ format were uploaded to the NCBI Sequence Read Archive (SRA) with SRA accession number PRJNA726299. The reference genome and gene annotation files were downloaded from EnsemblPlants release-32 (http://plants.ensembl.org/index.html). The paired-end clean reads were aligned to the reference genome using Hisat2 v2.0.5., and the mapped reads were assembled by StringTie (v1.3.3b). Fragments Per Kilobase of transcript sequence per Millions (FPKM) of each gene was calculated based on the length of the gene and reads count mapped to the gene. The genes with fold change ≥2 and corrected *P*-value < 0.05 in comparisons were considered as significant DEGs. The identified DEGs were then implemented to GO enrichment analysis and KEGG enrichment analysis.

### Proteomics analysis and data analysis

Three independent biological replicates were used, and at least 10 whole seedlings were mixed in each replicates in this research. Samples were grounded individually in liquid nitrogen and total protein was extracted by cold acetone method. The protein samples were then labeled by TMT tags. Shotgun proteomics analyses were performed using an EASY-nLC™ 1200 UHPLC system (Thermo Fisher) coupled with a Q Exactive™ HF-X mass spectrometer (Thermo Fisher) operating in the data-dependent acquisition (DDA) mode by Novogene Bioinformatics Technology Co. Ltd. (Beijing). Proteins with fold change in a comparison > 1.2 or < 0.83 and unadjusted significance level *p* < 0.05 were considered as DEPs. The DEPs were then analyzed by GO and KEGG enrichment analyses. The protein-protein interactions were predicted using STRING-db server (http://string.embl.de/). The mass spectrometry proteomics data was deposited to the ProteomeXchange Consortium (http://proteomecentral.proteomexchange.org) via the iProX partner repository [38] with the dataset identifier PXD025824.

### Correlation analysis between proteomic and transcriptomic results

The DEGs and the DEPs were separately counted, and the Venn diagrams were plotted according to the counted results. Correlation analysis was performed for the differential multiples of DEGs or DEPs identified in both transcriptomic analysis and proteomic analysis by R (version 3.5.1). The collected DEGs and DEPs in correlation analysis were also analyzed by GO and KEGG enrichment analyses.

### qRT-PCR analysis

10 DEGs were selected randomly for qRT-PCR verification, and the CDS of selected 10 DEGs were listed in the Supplementary material 8. The primers listed in Supplementary material 9. In the comparisons of Se accumulation related genes in different Se content cultivars, 5 genes related with Se uptake and transportation including *TaSBP1*, *TaOASL*, *TaHMT*, *TaCS* and *TaSultr1;3* were selected, and the basic information about these genes including gene names, accession numbers, their functions, primers were listed in the Supplementary material 7. The qRT-PCR program contains a preliminary step of 2 min at 50 °C, 10 min at 95 °C, followed by 40 cycles of 95 °C for 60 s, 56 °C for 20 s, and 72 °C for 15 s. Three independent biological replicates and three technical replicates were used. Primers were designed using Primer Premier 5.0 (Premier), and the TaACTIN (GenBank: AB181991) was used as the endogenous control. The primer efficiency was tested by generating standard curves, and the data were analyzed by the comparative ΔΔCT method.

### Se concentration detection

The Se concentration in the dried wheat seeds were detected by AFS-933 atomic fluorescence photometer (Beijing Jitian Instrument Co., Ltd). In details, the dried seeds were grinded and fined through 1 mm sieve, and 1 g samples were used and digested by mixture of 10 ml HNO_3_ and 2 ml HClO_4_ overnight. Then heated in 140 °C until the sample solutions turned into light yellow color or colorless. After get rid of acids in the sample solutions, the samples were detected by AFS-933 atomic fluorescence photometer.

### Physiological indexes detections

In this research, 2-week-old wheat seedlings of cultivars JM22, LY502, and QM6 were sprayed with 0, 1, 5, 10, and 20 μM Na_2_SeO_4_ for 2–3 days, respectively. The threated seedlings of bread wheat were used for physiological indexes detections. The nitrogen content and relative content of total chlorophyll were measured by PJ-4 N plant nutrition analyzer, and the relative permeability of cell membrane, damage rate of leaves, MDA content, SOD activity, POD activity, and CAT activity were detected as previously described (Lu et al., 2011).

### Statistical analyses

Statistical analyses were performed by SAS, and the statistical significance of the difference was evaluated by ANOVA. Means followed by the same letter were not significantly different at α = 0.05 level.

## Supplementary Information

**Additional file 1: Supplementary material 1** Different Se accumulation in divergent wheat cultivars.

**Additional file 2: Supplementary material 2** The total gene difference between groups was more significant than the variability among three replicates in a group by PCA analysis. Different groups were donated by different colors.

**Additional file 3: Supplementary material 3** The DEGs annotation in the Se responses of bread wheat.

**Additional file 4: Supplementary material 4** The total protein difference between groups was more significant than the variability among three replicates in a group by PCA analysis. Different groups were donated by different colors.

**Additional file 5: Supplementary material 5** The DEPs annotation in different comparisons.

**Additional file 6: Supplementary material 6** The DEPs/DEGs annotation in the correlation analysis between proteome and transcriptome.

**Additional file 7: Supplementary material 7** The basic information about the genes related with Se uptake and transportation in qRT-PCR.

**Additional file 8: Supplementary material 8** The sequence templates of randomly selected DEGs in qRT-PCR confirmation.

**Additional file 9: Supplementary material 9** Oligonucleotide primers used in qRT-PCR confirmation.

## Data Availability

The mass spectrometry proteomics data have been deposited to the ProteomeXchange Consortium (http://proteomecentral.proteomexchange.org) via the iProX partner repository with the dataset identifier PXD025824. The FASTQ files of raw data were uploaded to the NCBI Sequence Read Archive (SRA), and the SRA study accession is PRJNA726299.

## Abbreviations

APGI: Archaeal phosphoglucose isomerase
APR: APS reductase
APS: ATP sulfurylase
CAT: Catalase
CPC: Carboxypeptidase C
CS: Cysteine synthase
CYP: Cytochrome P450
DDA: Data-dependent acquisition
DEG: Differentially expressed gene
DEP: Differentially expressed protein
FPKM: Fragments per kilobase of transcript sequence per millions
GO: Gene ontology
GR: Glutathione reductase
GRX: Glutaredoxin
GS: Glycogen synthase
GSH-Px: Glutathione peroxidase
GSS: Glutathione synthetase
GST: Glutathione S-transferase
H_2_O_2_: Hydrogen peroxide
HSP: Heat shock protein
KEGG: Kyoto encyclopedia of genes and genomes
LOX: Liposygenase
LRR: Leucine-rich repeat
MDA: Malondialdehyde
Me-SeCys: Methyl-SeCys
MS: Malate synthase
Na_2_SeO_4_: Disodium selenate
P: Phosphorus
PCA: Principal component analysis
PO_4_^3^: Phosphate
POD: Peroxidase
ROS: Reactive oxygen species
PR1: Pathogenesis-related protein1
qRT-PCR: Quantitative real time PCR
Se: Selenium
SeCys: Selenocysteine
SeMet: Selenomethionine
SeO_4_^2^: Selenate
SeO_3_^2^: Selenite
SO_4_^2^: Sulfate
SOD: Superoxide dismutase
SRA: Sequence read archive
Suc-6-PH: Sucrose-6-phosphate hydrolase
TMT: Tandem mass tag-based
TrxR: Thioredoxin reductase
UFGT: Udp-glycose flavonoid glycosyltransferase
UQ: Ubiquinone
Xyn: Endo-1,4-beta-xylanase
