# Supplementary figures and images for: Transcriptome and proteome profiling revealed molecular mechanism of selenium responses in bread wheat (Triticum aestivum L.)
Source: BMC Plant Biol. 2021 Dec 9;21:584. doi: 10.1186/s12870-021-03368-w (PMC8656055; doi:10.1186/s12870-021-03368-w)

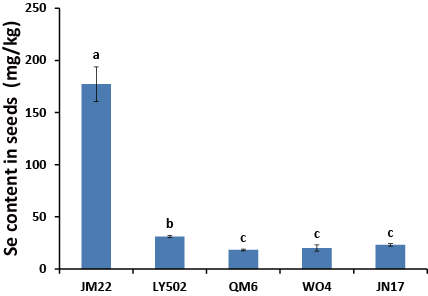

Supplement: Supplementary file 1 — Additional file 1: Supplementary material 1 Different Se accumulation in divergent wheat cultivars. [file 12870_2021_3368_MOESM1_ESM.png]

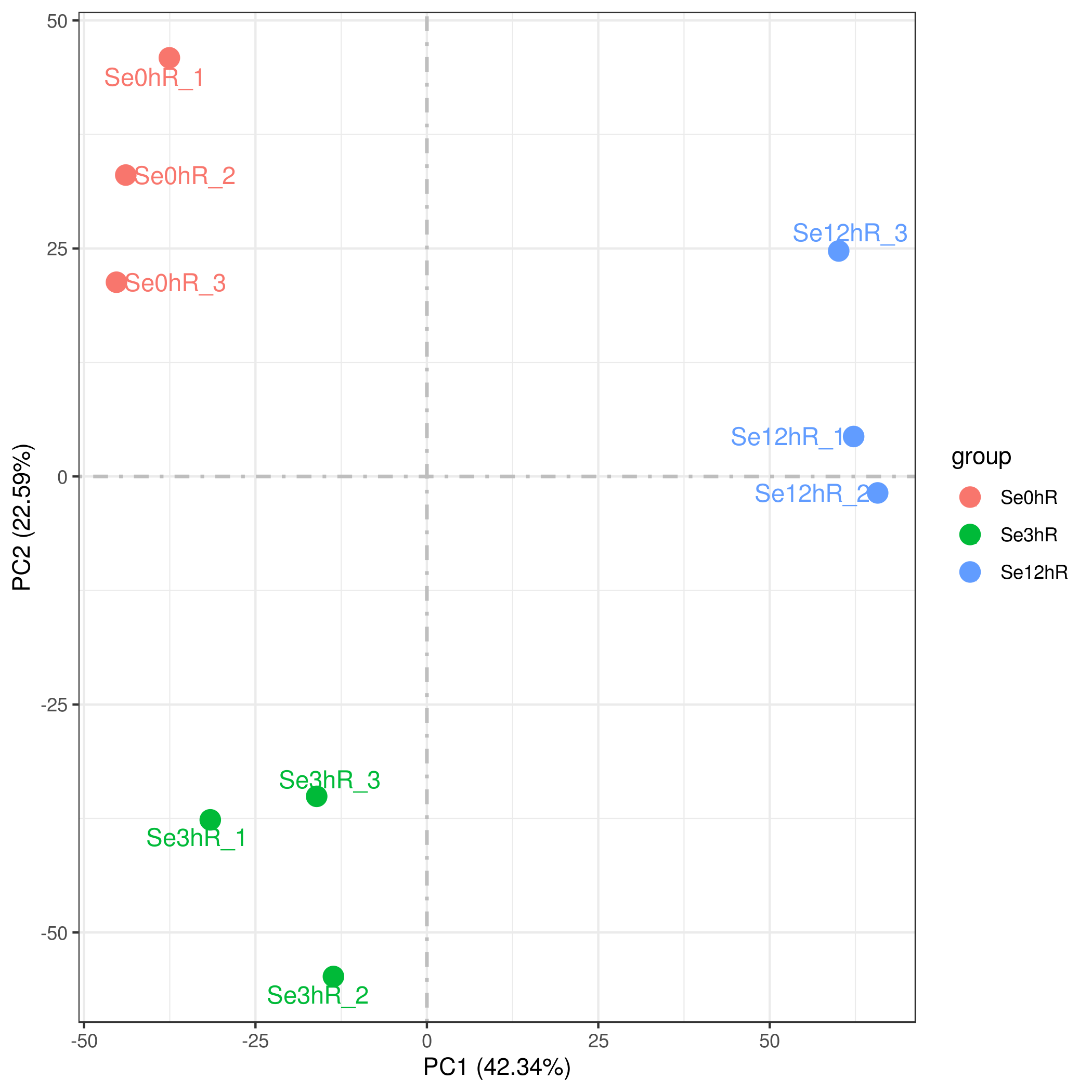

Supplement: Supplementary file 2 — Additional file 2: Supplementary material 2 The total gene difference between groups was more significant than the variability among three replicates in a group by PCA analysis. Different groups were donated by different colors. [file 12870_2021_3368_MOESM2_ESM.png]

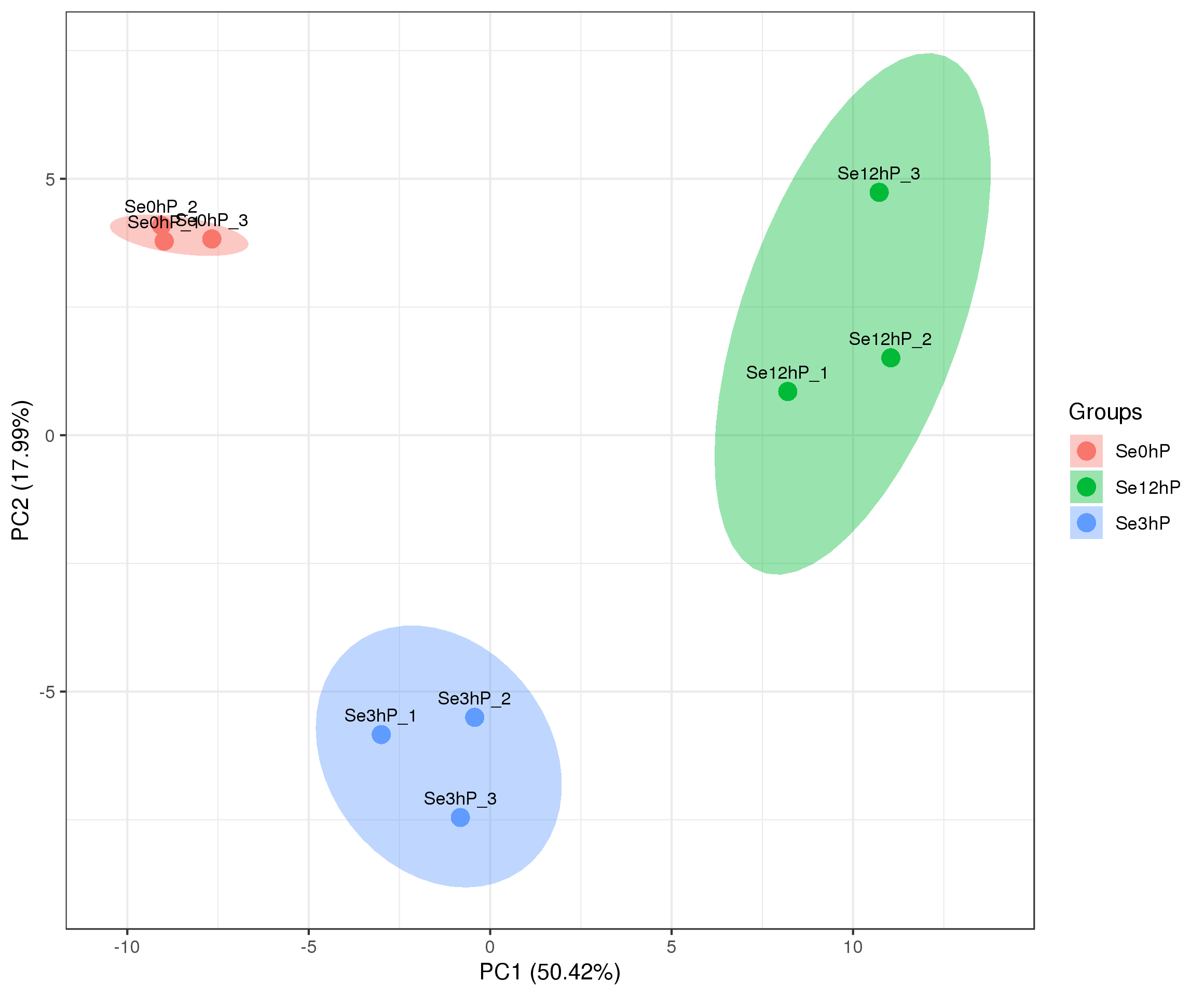

Supplement: Supplementary file 4 — Additional file 4: Supplementary material 4 The total protein difference between groups was more significant than the variability among three replicates in a group by PCA analysis. Different groups were donated by different colors. [file 12870_2021_3368_MOESM4_ESM.png]
