# Supplementary material for: Transcriptome and proteome profiling revealed molecular mechanism of selenium responses in bread wheat (Triticum aestivum L.)
Source: BMC Plant Biol. 2021 Dec 9;21:584. doi: 10.1186/s12870-021-03368-w (PMC8656055; doi:10.1186/s12870-021-03368-w)
Supplement: Supplementary file 7 — Additional file 7: Supplementary material 7 The basic information about the genes related with Se uptake and transportation in qRT-PCR. [file 12870_2021_3368_MOESM7_ESM.docx]

Supplementary material 7: The basic information about the genes related with Se uptake and transportation in qRT-PCR.

| gene name | accession number | function | Primer-F (5’→3’) | Primer-R (5’→3’) |
| --- | --- | --- | --- | --- |
| *TaSPB1*  *TaCS*  *TaOASL*  *TaHMT*  *TaSultr1;3* | TraesCS3A02G422100  TraesCS3D02G332100  TraesCS3B02G102500  TraesCS5A02G053100  TraesCS4D02G264100 | Selenium binding protein  Cysteine synthase  O-acetylserine lyase  Homocysteine methyl transferase  Sulfate transporter | AGGTCACAGCCCCTTGTTTG  GCTGCTGCTAGAGGATACAA  CTTGCTTTCATTGCTGCTTCCA  CTACCATTCAAGGGTTTGAGTCCA  AGTAGCTTCGTTCCGCCTTT | TGCTCCCTAGATCCAGTGTCT  GCTGGATTGTTGAATTGCTGA  AACTGCTCAAGCATGTATGAATTGG  GTACTCAGAGCCATCAGCAAG  AATCCAAGCATCGCCTGAGT |
