# Supplementary material for: Transcriptome and proteome profiling revealed molecular mechanism of selenium responses in bread wheat (Triticum aestivum L.)
Source: BMC Plant Biol. 2021 Dec 9;21:584. doi: 10.1186/s12870-021-03368-w (PMC8656055; doi:10.1186/s12870-021-03368-w)
Supplement: Supplementary file 8 — Additional file 8: Supplementary material 8 The sequence templates of randomly selected DEGs in qRT-PCR confirmation. [file 12870_2021_3368_MOESM8_ESM.docx]

>TraesCS4B02G325800 (*TaCAT*)

ATTCTGGCCGGGGAGCGCCCGCCGGGGCGCGTGTGGATGAGGCGCGCGCGGCCCGTATCCGCCCACCCGACCCCCGGAGCAGGCAGAAGCACACGTACGCCCACGAACGATCGAAGGCCCAGAAGCGTCGATCCGGCACGGGGACAAATCTCTACGGTCCTCTCGTTCCCGCTATAAAACTATCGGCGCCGCGGGGCGAGGCTCGCCTAGTCGTCATTCCGCGAGCGCACCGCCGGCCGTCCGCCCGCCGACCGTTCCTCCGTTCGCCATGGACCCCTACAAGTACCGGCCGTCGAGCTCCTTCAACGCCCCGATGTGGAGCACCAACTCCGGCGCGCCCGTCTGGAACAACGACAACTCCCTCACCGTCGGATCCCGAGGTCCGATCCTGCTGGAGGACTACCACCTGGTGGAGAAGATCGCCGACTTCGACCGTGAGCGCATCCCGGAGCGGGTGGTACACGCCCGGGGCGCCAGCGCCAAGGGCTTCTTCGAGGTCACCCACGACGTCTCCCACCTGACCTGCGCCGACTTCCTCCGCGCGCCGGGGGTGCAGACCCCCGTCATAGTGCGCTTCTCCACCGTGATCCACGAGCGCGGCTCCCCCGAGACGCTCCGCGACCCGCGCGGCTTCGCCATCAAGTTCTACACCCGGGAGGGCAACTGGGACCTGGTGGGCAACAACTTCCCCGTCTTCTTCATCCGCGACGGCATGAAGTTCCCGGACATGGTGCACGCGCTCAAGCCCAACCCCAAGACCCACATCCAGGAGAACTGGCGGATCCTCGACTTCTTCTCCCACCACCCGGAGTCGCTCCACATGTTCACCTTCCTCTTCGACGACATCGGCGTGCCCGCCGACTACCGCCACATGGACGGCTCCGGCGTCAACACCTACACGCTGGTGAACCGCGCCGGCAAGGCGCACTACGTCAAGTTCCACTGGAAGCCCACCTGCGGCGTCAAGTCGCTGCTGGAGGAGGAGGCGGTCACGGTGGGCGGCACCAACCACAGCCACGCCACCAAGGACCTCACCGACTCCATCGCCGCCGGCAACTACCCGGAGTGGACCTTCTACATCCAGACCATCGACCCGGACTATGAGGAGCGGTTCGACTTCGACCCGCTGGACGTGACCAAGACGTGGCCCGAGGACGTGGTGCCGCTGCAGCCCGTGGGGCGGCTGGTGCTGAACCGCAACATCGACAACTTCTTCTCGGAGAACGAGCAGCTGGCCTTCTGCCCCGGGATCATCGTCCCCGGGGTGTACTACTCGGACGACAAGCTGCTGCAGACGAGGATCTTCTCCTACTCCGACACGCAGCGCCACCGTCTGGGGCCCAACTACCTGCTGCTGCCGGCCAACGCGCCCAAGTGCTCCCACCACAACAACCACTACGACGGGCTCATGAACTTCATGCACCGCGACGAGGAGGTCGACTACTTCCCCTCAAGGTTCGACCCCGCCAAGCACGCGCCCCGCTACCCCATCCCCTCCCGCACCCTCAACGGCCGCCGCGAGAAGATGGTGATCGAGAAGGAGAACAACTTCAAGCAGCCCGGGGAGAGGTACCGCTCCATGGACCCGGCAAGGCAAGAGCGATTCATCAACAGATGGATCGACGCGCTGTCGGACCCCCGCCTCACCCATGAGATCAAGGCCATCTGGCTCTCCTACTGGTCTCAGGCTGACAAGTCTCTCGGCCAGAAGCTCGCGAGCCGTCTCAGCTCCAAGCCGAGCATGTAAGATCAGTCCATGGAGTGCGCAAATCAGAAATCTGCTTCAGGGCCGTCCAAGTTCTTCCCTCTTATCGGCAAAGAATTACGTACCAAATGCTGCATCTACCTACTTCATTTTACTGAGCCTATTTTGAAAAGAGGACATACATATGATTCTACCACACGTTATGCGCATTGTCGTCCGTCATTCATGTAATAATTTGTACAAGAGCCCGTTTCTCCTGATACTTCATGATTATTGTAAAGTGAATGCATCTTGATTAATGAACCAAATTAAGAATAAGTTGAAGAGTATGTATACCAATAATCTCTTGTGTTTGTCCTTT

>TraesCS6D02G154600 (*TaLOX*)

CAGAGCACTTGGGAGTGACGAGAGAGAGGCCGGCCCTCCTAGCTAGGTAGCTGACTAATCAGAAGAAGAAAAGAAGGTAGAGGTCGAACCAGCTCGATCCATCGGCAGCAGCTAGCTAGCCCTGCCCGCGCCAGCCATGATCCATCTGAAGCAGCCGCTGGTGCTCTCCGCGCAGAGCAGCAATGTTGCCTCACCGCCGCTCTTCGCCACGGCGGCGGCCGCGAGCGGCCAGCAGAGGCGGGCGTCCGGCGCCGGGAGGAGCCGCTCTGGGCGCCGGCTCACGGCGCGCAGGATAAGCTGCGCGTCGACCGAGGAGGCCGTCGGCGTCTCGACGTCCGTGACGACCAAGGAGAGGTCGCTGACGGTGACGGCCGTCGTGACCGCGCAGGCGCCGACCTCCGTGTACGTCGCCCGCGGCCTCGACGACATCCAGGACCTCTTCGGCAAGACACTGCTGCTCGAGCTCGTCAGCTCCGAGCTTGACCCCAAGACGGGAAGGGAGAGGGAGAAAGTTAAGGGGTTCGCGCACATGACGCTCAAGGAGGGGACGTACGAGGCCAAGATGTCGGTGCCGGCGTCGTTCGGGCCGGTGGGCGCGGTGCTGGTGGAGAACGAGCACCACAGGGAGATGTTCATCAAGGACGTCAAGCTCATCACCGGCGGCGACGAGAGCACCGCCATCACCTTCGACGTCGCCTCCTGGGTGCACTCCAAGTTCGACGACCCCGAGCCGCGCGTCTTCTTCACCGTCAAGTCATACCTGCCGTCGCAGACGCCGCCGGGAATCGAGGCGCTGAGGAAGAAGGAGCTGGAGAAGCTGCGTGGCGACGGGCACAGCGAGCGCAAGTTCCACGAGCGCGTCTACGACTACGACACGTACAACGACCTCGGCGACCCTGACAAGAACATCGACCACAAACGCCCGGTGCTGGGCACCAAGGAGCACCCCTACCCTCGCCGGTGCCGCACCGGCCGGCCCAAGACCCTCTACGATCCGGAGACGGAGAGGAGGAGCTCGCCGGTGTACGTGCCGCGCGACGAGCAGTTCTCGGACGTCAAGGGGCGGACGTTCAGCGCGACGACGCTGCGGTCGGGGCTGCACGCCATCCTGCCGGCGCTGGCGCCGCTGCTCAACAACTCGCAGTGCTTCTCGCACTTCCCGGCCATCGACGCCCTCTACAGCGACGGCATCCCGCTCCCCGTCGACGGGCACGGCGGCGCCTCCTTCAACGTCATCAACGACGTCATTCCCCGTGTCGTCCAGATGATCGAGGACACCACCGAGCACGTCCTCCGCTTCGAGGTCCCCGAGATGCTTGAGAGGGACCGATTTTCGTGGTTCAGAGACGAGGAGTTCGCGAGGCAGACGCTCGCCGGGCTCAACCCTATCTGCATCCGCCGCCTCACGGAATTCCCCATCGTGAGCAAGCTGGACCCGGCGGTGTACGGGCCGGCGGAGTCGGCGCTGACCAAGGAGGTCCTGGAGAAGATGATGAACGGGCGCATGACGGTGGAGGAGGCGGTGGAGAAGAAGCGGCTGTTCCTGCTGGACTACCACGACGTGTTCCTGCCGTACGTGCACAGGGTGCGCGAGCTGCCGGACACGACGCTGTACGGGTCCCGCACCGTCTTCTTCCTGAGCGAGGAGGGCACGCTGATGCCGCTGGCCATCGAGCTGACGCGGCCGCAGTCGCCGACCAGGCCGCAGTGGAGGCGCGCCTTCACGCACGGCCCCGACGCCACCGAGTCGTGGCTGTGGAAGCTGGCCAAGGCGCACGTGCTGACCCACGACACCGGCTACCACCAGCTGGTCAGCCACTGGCTGCGCACGCACGCCTGCGTCGAGCCCTACATCATCGCCACCAACCGGCAGCTCAGCCGGATGCACCCGGTGCACCGCCTGCTGCACCCGCACTTCCGCTACACCATGGAGATCAACGCGCTGGCCAGGGAGGCGCTCATCAACGCCGACGGCATCATCGAGGAGGCCTTCTGGCCGGGGAGGTACTCCATCGAGCTCAGCTCCGTCGCCTACGGCGCCGCCTGGCAGTTCGACACGGAGGCGCTGCCGGAGGACCTGGTAAGCCGGGGGCTCGCCGTGCGCAGGGACGACGGCGAGCTCGAGCTCGCCATCAAGGACTACCCGTACGCCGACGACGGGCTCCTCATCTGGGGCTCCATCAAGCAGTGGGCGTCCGACTACGTGGACTTCTACTACAAGTCGGACGGCGACGTCGCCGGCGACGAGGAGCTGCGGGCGTGGTGGGAGGAGGTGCGCACCAAGGGGCACGCGGACAAGAAGGACGAGCCGTGGTGGCCCGTGTGCGACTCCAAGGAGAACCTCGTCCAGATCCTGACCATCATCATGTGGGTCACGTCCGGCCACCACGCCGCCGTCAACTTCGGGCAGTACCATTACGCCGGGTACTTCCCCAACCGTCCGACGGTGGTGCGGAAGAACATCCCGGTGGAAGAAAACCGGGACGATGAGATGAAGAAGTTCATGGCAAGGCCAGAGGAGGTGCTGCTGCAGAGCCTCCCCTCGCAGATGCAGGCCATCAAGGTGATGGCGACGCTGGACATCCTCTCCTCACACTCCCCCGACGAGGAGTACATGGGAGAGTACGCGGAGCCGGCGTGGCTGGCAGAGCCCATGGTGAAGGCGGCATTCGAGAAGTTCAGTGGCAGACTGAAGGAGGTGGAGGGCACCATCGACCAGCGAAACAACAACCCGGAGAACAAGAACAGGTGTGGCGCCGGCATCGTGCCGTACGAGCTGCTCAAGCCGTTCTCAGAACCAGGGGTCACTGGGAGGGGCATCCCCAACAGCATCTCCATCTGAATGTGGCCCCAGGATAAGTTATCATTGCATGTAGGATTAGGAATAAGATGTGCACCATGTACGCACCCAGCAGCGATTGCTCCCGTCCCGTGCATGACAGGGGCCAGGAGAGGCATCTACTAGTGTGTTCCCGCTATTGTATGCCGTGAATTAGCAGTACTAATAAGGTTATTTGAATTGTGACCTCATCGAATCCTAATTAGGTTAAGAAAGAGTGGGCATCTACATGCATTCCAGGACAGTCATACTGTACCGTATCATTTGGAGCATGCCATACAAAACAAAACAATCACAT

>TraesCS5A02G325200 (*TaUDPGT*)

CACATTCACACGCCGATCCACCGCCACCTGGAGCGATACTGCTAGCTACGACCATGGACGCCAACGCCGATGCCCGCTCCTCGTCCTCGCCCATGCACATCGTCATCTTCCCGTGGCTCGCGTTCGGGCACATGATCCCCTTGCTGGAGCTTGCAGAGCGCCTGGTGGCGCGCGGCCACCGCGTCTCCTTCGTCTCCACGCCGCGCAACCTCAGCCGGCTCCGGCCGGTCGTCGGCGTCCACTTCGTCGCCCTGCCGCTGCCCCGCGTGGACGGCCTCCCGGAGGGAGCCGAGGCCACCTCCGACCTCCCGCCCAGTCCCGGCAACCTGGCCGAGCTTCTACTCAAGGCCGCCGACGGCCTCGTCGGCCCATTCTCCGCCTTCCTCGACGAGGGCAAGAAGCCAGATTGGCTCGTCCTCGACAACCTCCACTACCTGGCAGCGGCCGCCGCCGCCGACCGAGGCGTGCCGTCCGTGATGTTCCTCCCCTTCGCCGCCGCGTCGACCGCGCTCTGGGGCGTGCCGCGTGTGTCCACGGTGGTGGACCCAGAGCTAGGGGCGACCGTGCCCCAGCGCTTCGTGCTAACCTACCAGTGCTGCAAGATCGTCGCCCAGCGGTGCTGCGTGGAGTTCGACCCCGAGGCCGTGCCTCTGCTGCCGGGCGTCCTGGGCAAGCCGTTCGCCCCTATGGGCCTGCTGCCGCCGCCGCCTCTGAGGGCGAGTTCGAATAATGAAGGTGACGAGCTCGTGTCATGGCTGGACCGGCATCCGGCAAAATCCGTCGTCTACGTCGCGCTGGGAACCGAAGCGCCGCTGACCACCGAGCTGGTGCACGAGCTGGCCATCGGCCTGGAGCTCGCCGGTACGCCGTTCCTGTGGGCTCTGAGGAAGGTCGGCGACCAAGACGTTCTTCCTCCGGGCTTCGAGGAGCGCATCAAGGGCCGCGGCCTCGTGGCGATGGGGATGGTTCCGCAGACCAGGGTGCTAGCGCACGGCTCCGTGGGCGCGTTCCTCACGCACAGCGGGCCGGGCTCCGCCATCGAGGGGATCCAGTATGGGCATCCCCTCGTCATGCTGCCCTTCTTCGGAGACCAACAGACGGGGGCTCAGTTCATGGAGAGGAAGAAGGTCGGTCTGCTGGTGCCGAGGAACGGGGAGGATGGACTGTCTTTTGACCGGCAAGGCGTCGCGTCCACGGTCCGGGCCGTCGTGGTGGACGAGCAAGCAGGACGCGTCTTCGCCGCCAATGCCAAGAAGTGGCAGCAGGTTGTCGCGGACACTGCCTGCCATGAGAGGTACATCGATGAGTTCGTTCAGCAACTCCGATTCTACAAGGACCAGTGAAGTGACTCCATGGCTAATGGAGGCGACAATGCATGACTCGTGCGTACGTGCATCACCCTCCGTCGAGTCGGAGATTCGTTCCACGTTTTATGGTTTCGGCAAAGTTTGGCTTCTTCCGTAATGTTGCTGTTAGCACGGTGTGTCATGTGTGCGTGTTCATGCTCACCGTGCACGTTCTCTTGAGTACGTCTTTCGAGTTATGCTGTGAAAATAAACTCATGTGAAAATCGGTCCTACCCCAAGTTAATAAGCCAACAAATTCTTTTCACTAC

>TraesCS6B02G051800 (*TaAT*)

GCTTTCCACCTCTGCTCGCCAAAAACAAGAGTCTCCTTCGCCCGCCGTCGCCATCGCCATCGCCCACGCCCGCCATCGCCATCGCCATCGCCACGCCAACCCCAGCAACCCGGCGTACAAATCCAGCGCGCATACCACTGGCCCCGGCCGCCCGGCACAATCATCGCGCGCTAGACCCACCGCCATCCGTCGGCCCATCGATCGATCTCCCTCCCTTCGTCCGTCCATGGCGCCGCGAGCGACGGTCGCGATGAGCAGGTTCCCGCCGGTGTCATCCTACGACGCGGCGGCGCGGGAGCGCCGCACCGCGGCCTCTGATCTGGACGGCACCCTGCTGGCCTCCTCCTCCGCGTTCCCCTACTACTTCCTCGTGGCCCTCGAGGCGGGCGGGTACCTCCGCGCCCTCGCGCTGCTCCTCCTCGCCCCGTTCATCCTGCTCCTCTACAGCGCCGTCTCCGAGCCGGCCGCCATCGGGCTCCTCGTCTTCGCCACCTTCGCGGGGCTCCGAGTGCGGGACGTGGAGGCCGTGGCGAGGGGCGTTCTTCCGCGGCACTACGCCGCCGGGGTGCGCGCCGACTCGTGGGAGGTGTTCCGGGGGTGCGGCGCGGGCAGGCGGGTCGTGGTCACCGCGTCCCCGGCGGTCATGGTCGCCCCGTTCGTACGCGAGTTCCTCGGGGCGGAGGTGGCCGGGACGGAGCTCGGGACCTGCTGCGGGCGCTTCACGGGGCTCATCAGCGGCGGGGTGCTTGTGGCCGGGAGGAAGAGGGAGGTCGTGGAGAGGCTGTTTGCCGGCGGGGACATGCCGGACGTCGGGCTCGGCGACCGCGAGAGCGACCACGACTTCATGGCCATCTGCAAGGAAGCCTACATGGTGCCCACGAACAAGCGCGCGCCGCGCGCGGCCGCCGACGCTCTGCTGTCCAGCGTCGTCTTCCACGACGGCCGCCTGGTCCGCCGGCCAGACCCGGCGCAGGCGCTCTTCGCGTTGGTCTACCTCCCGGTGGGCTTCGCCCTGGCCGTCCTCCGCGTCCTCATCAGCCTCCCGGTCCCGCCGCACCTCGTGCGCCACACGTACCGCCTGACCGGCATCCAGCTCGCCGTGCGGGGCACGCCCCCGCCGGCGCCGCGCGAGGGCTCCCCCGGGTCCCTCCTCGTCTGCAACCACCGCACGGCGCTGGACCCCATCATCGTGGCCGTGGCGTTGGGGCGACCGGTGACGTGCGTGACCTACAGCGTGAGCCGGCTGTCGACGGCCATCTCGCCGATCCCGGCGGTGGCGCTGGCGCGGGACCGGGAGGCTGACGCGGCGCGCATCGCGGCGCTGCTGAACTCCGGGCGCGACGTGGTGGTGTGCCCCGAGGGGACGACGTGCCGGGAGCCCTGCCTGTTGCGTTTCTCGGCGCTGTTCGCGGAGCTGACGGACCGGATCGTGCCTGTGGCGTTGGAGGCGGCGCAGTCGACCTACTACGGGTCCACGGCGAGGGGATGGAAGGCCATGGACCCGTGGTTCTTCTACATGAACCCGCGGCCGGGGTACAAGGTGACGTTCCTGCCGGCGCTCCGGCCGGAGGAGACGTGCGGCGCCGGCGGGAGGAGCGCCGTGGACGTGGCCAACCACGTGCAGGCGGTGATCGGCAAGGAGCTCGGGTACCGGTGCACCACGCTCACCAGGAAGGACAAGTACATGAAGCTCGCCGGCAACGACGGCACGGTCGCCGCCGACGGCGACGACGGCAAGAAGCTTGCGTAAACCGCGGAACTGTATAGGTAGTCACGATTCATGGACCAGCATAGTGATGTAGTTTCTGTTCTGTAGGCTGTACCCCGATTTTCTAGTGTTTTCCCCTGCTTTGGCAATTTCACTTACCCTGCACATTGTCTGGTCAAACAGAAGAAAGTTGACAGCAACAGCAAGCATGAACATCAAGATAGAGTCCAGATGGAGATTGATAGTTTTCCTTTTTCATTTATTAGTTCAACAGTTTCTACTAAAGAACATTTTTCC

>TraesCS6D02G177400 (*TaST*)

GGGGTAGCTCTTGGCTCCTATTTGAGGCGGCTTCGCTCGGTTCTGATCTACCGCACCACACCACCACACCACACCAGGGGCCTGCCGCTTCTTGGGCTTCTCCATCTCATCTCCTTGGCGCAGCTGCAGGGATCCTTGGTGGAGAGGAGGGAAGAAGATGTCGGGTGCTGCACTGGTCGCGATTGCGGCTTCCATTGGCAATCTGCTGCAGGGGTGGGACAATGCCACCATCGCTGGTGCTGTTCTGTACATCAAGAAGGAATTCCAGCTCGAAAATAATCCGACTGTGGAGGGGCTCATCGTGGCCATGTCGCTCATCGGTGCAACCATCATCACCACATTCTCCGGGCCAGTATCAGACTGGGTTGGCCGGCGCCCTATGCTCATTCTCTCTTCGCTTCTCTACTTCCTCAGCGGCTTAATCATGCTATGGTCACCCAATGTCTATGTGCTGCTCCTGGCACGCCTTGTCGATGGCTTTGGTATCGGCTTGGCTGTCACACTGGTGCCTTTGTACATCTCAGAGACGGCTCCTTCGGAGATCAGGGGAAGGCTCAACACGCTTCCACAGTTCAGTGGGTCAGGAGGGATGTTCTTGTCATACTGCATGGTGTTCGGGATGTCACTCTTGCCATCACCTGATTGGAGAATTATGCTCGGGGTTCTCTCAGTTCCGTCGTTGTTTTTCTTCGTCTTGACGGTATTTTACCTGCCAGAATCTCCGAGATGGCTTGTCAGCAAGGGCCGAATGGCAGAGGCAAAGAAGGTGCTGCAAAGATTACGGGGAAGGGAGGATGTCTCAGGAGAAATGGCCCTTCTTGTTGAAGGTTTGGAGGTTGGAGGAGACACCTCCATTGAGGAGTACATAATAGGACCAGCTAATGACCCAGCTGATGATCATGTTGTTGATGGCGATAATGACCAAATAACACTATATGGGCCTGAAGAGGGCCAATCATGGATTGCTCGACCTTCCAAGGGACCCAGCATGCTTGGAAGTGTGCTTTCTCTTGCATCTCGTCATGGTAGCATGGTGAACCAGAGTGTGCCCCTTATGGATCCTCTAGTCACGCTTTTCGGAAGTGTTCATGAGAACATGCCTCAAGTTGGAGGAAGCATGCGGAGCACATTGTTTCCTAACTTTGGCAGCATGCTCAGTGTGGCAGATCAGCACCCAAAAACTGAGCACTGGGATGAGGAGAACGTTCATAGGGACGATGAAGAATATGCATCTGATGCTGGAGGCGACTACGAAGATAATGTCCATAGCCCGCTGCTGTCACGACAGACCACAAACACGGATGGGAAGGACCATGGTCACCATGGAAGCACTTTGGGCATGAGAAGGAGAAGTCTCTTGGAAGAGGGTGGGGAGGCAGTCAGCAGCACTGGTATTGGTGGGGGGTGGCAACTCGCATGGAAATGGTCGGAGCGACAAGGCGAGGATGGCAAGAAGGAAGGAGGCTTCAAAAGAATCTACTTGCACCAAGAGGGGGTGGCCGACTCAAGAAGGGGCTCTGTTGTTTCACTTCCTGGTGGGGGTGATGCCACGCAAGGGGGCAGTGGGTTTATACACGCAGCTGCTTTGGTAAGCCACTCGGCTCTTTACTCCAAGGATCTTATGGAAGAGCGTATGGCGGCTGGTCCAGCCATGATTCATCCATTGGAGGCAGCTCCCAAAGGTTCAATCTGGAAAGATCTGTTTGAACCTGGTGTGAGGCGTGCATTGTTCGTCGGCGTTGGAATTCAGATGCTTCAGCAGTTTGCTGGAATAAATGGAGTTCTCTACTATACTCCTCAAATTCTGGAGCAAGCTGGTGTGGCGGTTCTTCTTTCCAATCTTGGCCTCAGTTCAGCATCAGCATCCATCTTGATCAGTTCTCTCACCACCTTACTCATGCTCCCAAGCATTGGTGTAGCCATGAGACTTATGGATATATCTGGAAGAAGGTTTCTGCTACTAGGCACAATTCCCATCTTGATAGCATCCCTAATTGTTTTGGTTGTGTCCAATGTTATCAACTTGAGTACGGTGCCCCACGCTGTGCTCTCCACAGTTAGCGTCATTGTCTACTTCTGCTGCTTTGTCATGGGCTTTGGCCCGATCCCCAACATTCTATGTGCAGAGATTTTCCCCACCAGAGCCCGTGGTGTCTGCATCGCTATTTGCGCCCTCACATTCTGGATTTGTGACATTATTGTTACCTACAGCCTGCCTGTGATGCTGAATGCTATTGGTCTAGCAGGTGTCTTTGGTATATATGCAGTCGTTTGCTGCATCGCCTTTGTGTTCGTCTACCTAAAGGTCCCAGAGACAAAGGGCATGCCCCTCGAGGTCATCACCGAGTTCTTTGCGGTTGGGGCGAAGCAAGCGCAGACCACCATTGCCTGATTCATCATGGAGCTTTGTTTTCAGTTTGCACACTGCGCTGAAAATTGCAAATTGGACGGGTCCTCGTGAGGAACGGAAAAACTTTTGAGTTGTAAATGAGATAGCTACCCAAAGAGTTCATCACGAGGAACGGAAGCTGTAAAAGTAGGAGGATTTCATGCCCCCATTTCATCGTCTATTCTTGCTTATTAGTACTGTACTGTAATCGTCATTAGTTGCTGTAGGGATGTTCAACTTGCTAATCTGATTCTGAACTACTATGCTGATGTCCGAAATAAAGAAAAAGCATGTTTTTTTTGTGT

>TraesCS3B02G334300 (*TaGST*)

AGATCGGGTCGTAACAGGGAGTGGCGATGGCTCCGGTGAAGCTGTACGGCATGACCATGTCGTGGAACGTGACGAGGTGCGTGGCGGCCCTGGAGGAGGCAGGCGTCGAGTACGACGTCGTGCCCATCGACTTCGGCGCCGGCGAGCACAAGACCCCCGACCACCTCGCCAGGAACCCCTTTGGTCAGATGCCGGTTTTGCAGGATGGTGACTTCTACGTCTGGGAATCGCGTGCTATTTGCAAGTACACATGCCGCAAGAACAATCCGGAGCTTTTGAAGGAGGGCAACCTCAAGGAATCAGCAATGGTAGATGTGTGGCTCGAGGTGGAGGCCCATCGGTACACGGCCGCAATGGAGCCTATTATCCTTGAGTGCCTCATCCGTCCTATGTTCGGGCGAGCCACTGACCAGAAAATTGTCGAGGATAACCTTGTGAAACTGAAGAAGGTGCTGGAGGTGTACGAGGCGCGCCTGACCAAGTGCAAGTACCTTGCTGGAGACTTCCTCAGCCTAGCGGACCTTAACCATGTGTCTATTACCGCATGCCTGGCGGCTACACCCTACGCTTCTTTGTTTGACGCGTATCTGCATGTGAAAGCCTGGTGGTCTGGTCTGATGGCGAGGCCGTCCGTCCAGAAGATCACTGCACTGAAGAAGCCGTATTTTAAGAATAGTGTCTAGCACAGTTTTCGCCGCGATCCACGAAATAAGCCGGGTTGTCTTCCGATGTTTGTCTGTCTAGGTGCGTGTTGGTGTAATAATTAGATAACACCCCGCGCGTTGCTGTGAGATTTTTTAGCAATAAACTGTGAAATGATTTGTTGCTAAAAGGTAAAAGAATCTAATGACGTGGA

>TraesCS3A02G457400 (*TaCULLIN*)

TCAGTCCCCCATCCGCCGTCGGCTGCCCTCCCCACCCCACCCCACGAGACGACGGCGGCGGCGACGACGGCGAGCAGCACCCGATCCTCTCCCCCTCCTCGGTAACCTCCTCCACGTCCTCTCCCCGCGCCCCTCCTGGTTGATTCCGCTTTTCCGTATCCGATCGATTCGTGCTCCGTTCCGAGCAGTCTGTTCGCTCCGCCGTGGGTCGGGAATCCGAATCCATCTGCCTGGTCCGTCCTCGAATGCGTCCGTCCCCGTCGGTCTGTTGCGAATCCCGTCGCCTGCTCGCCGTCGCTGCCTTGCTGAATGCCGCCGCTGCCGTAGGGTTTAGTTTTTTTTTCCTTTCGCCGCGGCGAGACCGACGGGGTCATCGAACCCTCGCCGCCGCGCGGCCTCTCCCCTCCCAGGCCTCCTCCGTCGAAGCACGGAAGCGGTTCTATCGTGTGGTCGCTGTTCCTCCCCTCTGGCCCGCTCTACCGGACTGGTGTGGAGTGGGGAGTGGGGAGGGGAGATTCACCCATCTAGTTCACTGCAGGAGCAAAGCAGTAAAAATGAATGTGCACCTCAGCTTTGAGGATGGCTGGAAGGTCCTGGAGCAGGGCATTGTGACATGTTCAAAGATTTTGGAGGGATCCACTGGTACAAGGCCTACTGTTGCTGAGTACATGAATTGCTATGACTGTGCTTATAGAATGGCGGTGCAGACAACCAGCTATTGTGAAGAGATGTACAATGGTTACAAGGCTACACTTGCAGAATCTGTTCGTGCACTGGTTTGCCCCCATCTGATGCACCAGAGAGATGGCTACCTTTTGAGACAGCTTGCGAAAATGTGGTCTAACTATTGTATCATGGTTAAATGTGTATCTGGCTTCTTCAACTATTTGGACCGTTGCTTTGTTGAGCAAAGAAAGCTACCTTGTCTTGAAGACACTGCTGCCACTTCTTTTTTCTCTACAGTCTTTTCCTTCTTCAGTCATGAAGTATCAGACGCTCTGCTGACTTCGATTCGCCAAGAGCGTGATGGAATCAATGCTGATATGGACATCTTGATGGGCATTATGCGTGGCATATGTCGCTCTGAAGTCAAATCCTTCATGAAAAATGCTGTTGTTCAAGATACATATGCTTACTACTCCAGGAAAAGTTCTGAATGGATTGTTCAGTACCCTCTACAAGATTACCTTGCCAAGGTTCAGGATTGTATGGAGAAGGAAACCATGAGATTGATGAGCTATCTGAGTATTGCTGAGGGTGATAGCACAGAGCTCTGTTTGAAGGTTGTTAGTGCTCCATTGATGCAAACTTATGACAGCTATGCAAGAGAGAAACAGATTGGTGGCCAACTTTTGCTTCAAACATACAAGACTGTAGAGGATGATCTGCTGGCCAGATGCAATCGCTTGACAATTGACAGCGGTGTGGACAACAGTTCTGGCAGTTACATGGAGTAGAAAGGAAATGTTCTTCAGCTAACATTCATCGATAACCAAGCTTACATACAGGAAGGGGTTCTAGAACATGGCCTACATCTGGGGGAATTTTTTGTGCGGTTCGGTTTTTGGTACCAAAGCAGAGCTCGTGTACTCTGGATTTTGTTGGCGAGGTTTGGCTAGATTAAACGCTATACTGAATCTGCAGGGGATACCTCTTTAGGGCCGCCTCCTACCCCATTATGGTTAACAGCATTTGAAATGTACATGATGGCGGGTCAATTTATGCCAATGGCAGTTTATTTTGATTGTCATCTAGCGAACATTAGGCCGGTCAGCACCTAGCACTCTTAGTGGCTGGACATGGAGTATGTCATGACACCAGAATGATTGGTGCATTGCCTGTGCCTTTGGCGTGCTTCTGATGCTGTCTAT

>TraesCSU02G207700 (*TaPC1*)

CTGTACCCAAAGGTAGCTAAACATCGTCCATATCGGACATGGCTTTCATGCGCTCGTCGTCCTCATCCATGGCTCTGGCTCTGCTGCCGCTACTGGTGGCAGCCGCATTCTTCATCCCCTCCATGGCTTCATCAGCTTCAGGTACCCTGGACCATGGCTTGGATGGTGAGGCACTGCTGATGCTGGGGAGGTTCCATGGGTGGATGGCGGCGCACGGTCGGTCGTACGCCACCGTGGAGGAGAAGCTGCACCGGTTTGAGGTGTACCGGAGCAACATGGAGTTCATCGAGGCGGCGAATCGGGACAGCCGGATGAGCTACAGCCTCGGCGAGACCCCGTTCACCGACCTCACCCACGACGAGTTCATGGCCATGTACAGCAGCAACGACGAGTCGTCATGGGAGTCGGAGGAGATGACGGTGATCACAACTCGCGCTGGCCCCGTCCACGAGGGCACCGCCGCCGTCGAAGAGCCACCTCGTCATACCAATACCAATCTGACGGCGGTGGTGCCTGCGAGCGTCGATTGGAGGGAGAAAGGCGTCGTCACAGCAGCCAAGTATCAAGGGGAATCCTGTTCGTCTTGCTGGGCGTTCACGTCGGTGGCGACGATGGAGAGCGCGCATGCGATCAGCACGGGCGGATCGCCACCGGTGCTGTCGGAGCAGCAGCTGGTGGACTGTCGGATTAACGGCTGCGGCAACAGTTGGATGGACAAGGCCTTCGAGTGGGTGATCCAGAACGGTGGCATCACCACGGAGGCGGCCTACCCCTACACCGGCAAGGTGGGCACGTGCCAAAGGGCCAAGCCGGTCGCGGTGAAGCTCAGAGGCTACAAGAAGATTTCACCACCCGGTGACGAGGCGGCGCTGATGGCGGCCGTGGCGCAGCAGCCCGTCGCCGCATCCTTCGACTACAGTGACCCCTGCTTCCAGCACTACATCCGCGGCGTGTACAACGCCGGTTGCTCCAGGTCGGGCGTGTACAACAAAGGGGCGTGCGGGACAACGCAGAACCACGCGCTGGCCATCGTCGGGTACGGGACCAAGCCTGACGGGACCAAGTACTGGATTGGCAAGAACTCGTGGACTGACCAGTGGGGCGACAAAGGCTTCGTCTATTTCCTTAGGGACTCGCCACCCTTGGGCTTGTGCGGCATTGCCAAGTACCCGCTTTACCCTATCATCTGATGATCCAGCCGCCGTGCACCTCCATCATCTTTGTGTGACTCCGTGCATCTCTACTTATATTATATATATGTAATGAATAATTGCACGGTACCTAGCTATTCAATTGTCTACTCCATGTCTATGCTATGCATGTAAAGGTTCGGGTGCTACACATGCATCCTTGCCTATAATAAATAAACAATCGTTTTGTTTTTTGTAGATTCATAGCTTTTGCTATGCACCTGACCTATATAATACATTATGTTTTACTATGTACGTATCTAAATAAAAAAATTAAAACGACTTGCAATTTGGGGTGGAGG

>TraesCS5A02G238300 (*TaGDSL*)

ATGGCCAACGCATTCCATCGGCAGCGACGCATGCTGTGCACTGCACAGGCTATAAATACAGCTACCCATGGACATACCATGCAGTGCAAGGCGCAGTCGATCGAGCAGCTACTAGCTAGCAGCGACCAAGCCAAGCTACACAAGGACATGGGTGCGCTTGGCCTCCTCCTCGTGATGGCAGCAGCCATGGTAGCGGCGGCGGCGGCAGCAGCAGCAACGGCGAGCGCTCCTCGCCCCTTCTTCGTGTTCGGCGACTCCCTGGTGGACAGCGGCAACAACAACTACCTGGCCACCACGGCGCGGGCCGACTCGCCGCCCTACGGCCTCGACTACCCGACCCACCGCGCCACCGGCCGCTTCTCCAACGGCCTCAACGTCCCTGACATCATCAGCGAGCACCTCGGGGCCGAGCCCGTGCTGCCCTACCTCAGCCCCCACCTCGACGGCCACAAGCTGCTCGGCGGCGCCAACTTCGCGTCCGCCGGCGTCGGCATCCTCAACGACACCGGCATCCAGTTTGTGAACATCATCCGTATCCAGAAGCAGCTGCGCTACTTCGAGCAGTACCAGAGCAGGGTGCGGCGGCTGATCGGCGAGCCTGCGACGCAGCGGCTGGTGCGGAGCGCGCTGGTGCTCATCACGCTCGGCGGCAACGACTTCGTCAACAACTACTACCTGCTGCCCTTCTCCGCCAGGTCCCGCCAGTTCGCGCTCCCGGACTACGTCCGCTACCTCATCGCCGAGTACAAGACCATCCTCCAGCAGCTCCACGGCCTGGGCGCCCGCCGCGTCCTCGTCACCGGCTCGGGCCCGATCGGCTGCGCGCCGGCGGAGCTCGCCACGCGGAGCGCCAACGGCGAGTGCGACCTGGAGCTGCAGCGCGCCGCCGCGCTCTACAACCCGCAGCTGGTGCAGATGACCAAGGACCTCAACGCGCAGTTCGGCGCCGACGTGTTCGTCGCCGTCAACGCCTACCGGATGCACATGGACTTCATCTCCGCCCCGGCGGCGTACGGCTTCGTCACGTCCAAGGTGGCGTGCTGCGGCCAGGGCCCGTACAACGGCGTGGGGCTGTGCACCGCCATGTCCAGCGTCTGCCCCGACCGCTCCCTCTTCGCCTTCTGGGACAACTTCCACCCCACCGAGAGGGCTAACCGCATCATCGTCAGCCAGTTCATGGCCGGCTCCCCGGACTACATGCACCCGCTCAACCTCTCCACCATCCTCGCCATGGACGCCGCCGCAAAGCCATAATCCACCATTACCCTACTGTTTTCTCTATCTCACTGTGCTAGCGAACTAGCCAGTAGCATCCGTGCGCAATAAAAATTCTCTGCGTGATTTTTATTTATCATCCTCTGCCATGTCTAGCAGAGTATCCGTCCTCTTAATTCGACTGAGTAATCAATAAAAATAATAAAGCAACTATGTTATTTTTTGTTTAT

>TraesCS7A02G130000 (*TaPT*)

CCGTCGGCTCCCCACGCACACAACGGAACAGACAGCAGGAATCTTCGCGAGCTATCAGGACGTGTCGCGCCGCCCGTCCCCCGTCCCGTCCCCCTCCCCCGCCGTCGCTCTCGTCCTGCCGAGATTCCGCACGAGCCCAACTGCCAGCCAGTTCTCACCGCCACTCGCTGCCCCGCCCGCCCGCCCGCCACCCGCTACAAGATCGCCCGCCGCCTCTTCTCCTCTTCTTGCAAGCTGAGCCACTCGCATCCTCGGGCTCAGATCTCTCCTCCAAGAGCCCCCCCGGCGGCGCCCCTCCTCCGTCCCCGGACCGTCATCCCGCAGGTTTGAGACCGGTGCATTGCTTGGTTGACCCCTACTTGTGGAAATGCTTGATTCACTTATTAGCCATGACCCGAAGGTGTTGACTGAGAAGAAGCCTGATGAGATAATTGTGTCTGGTGTTGTTGAAAGTCTGCAGAATTTTCTACGCAAGTGCATCATAGCTGTCCTCTCATACGGCCCGATGCCTAAACACATCGCGTTTATTATGGACGGGAACCGCAGATACGCCAAGTCCAGAAGTATCAAGGAAGGCACCGGTCACAGCGTGGGCTTCTCTGCTCTAATGGCAAGTCTTATCTACTGTTACGAGATGGGTGTCAAGTACATCACGGTGTATGCATTCAGCATCGACAATTTCAAACGCGACCCTAGTGAGGTCAAGACCTTGATGGAATTAATGGAGGAAAAGATCAACGAGCTGTTGGAAAACAAGAATGTCATCAACAAGGTCAACTGCAAGATCAACTTCTGGGGGAACCTGGACATGCTTCCCGAACCAGTGCGGCTGGCAGCCCAGAAGCTGATGGCGAGCACCGCCGAGAACACAGGACTGGTCTTCTCCGTCTGCATGCCGTACAACTCAACCTCCGAGATCGCCAACGCCGTCACCGAGCTCTGCAAAGAACGGAGGGATATGATGCAGGGGCAGCAGGCCAGCGGCCGCAATGGCCGCCCCGCAAATGACGGCGCACGTTCGGATATCTCGGTGGCCGACCTGGATCACCACATGTACAGCGCCGGCTGTCCGGACCCGGACATTGTGATCCGGACCTCGGGCGAGACCCGGCTGAGCAACTTCCTCCTGTGGCAGACAACGTTTAGTCATCTGCAGAACCCGGACCCCCTCTGGCCTGAGTTCTCCTGGAGGCACCTCATCTGGGCGATACTGCAGTACCAGAGAGCCTACCCGTACATTGAGCAGAATAAAGGTCTGGCGAAGAAGCAGCTGTGAGTGAGGGATCTCATCTCCCTCGTAAGGTGATATATGCATATGATCATCTAGAAGATACTACAATAATAACAGATCAGAAGGCTGAGAAGCGTCAGTCTGTGAATAAGGGATCTCATCTCCTTTGTTAGGAGATAAGCAGATACCATGGAAAGATAGTAACAGCAGCCTGTTGAAAGTTGGCCCTTATTATTTTTCGTGTAATGAAGACTGTAATTATGGGAACCATGTGTATTGTTGTGTTAAACGGTGGCAATAGCTTGCCATTTAGCCTGAACTAAGAAGCCAATTCGAGAAAATTGTAAAGCGTCAGCGATATATGATCAGCGCTCATTAGTTCTCCAGGTAATTTGGGAGGGAGGGAGTATAATTTAAATTTGTCTCGTTTTTATAGCTGGAAGAAGCTTGTTGTGGTGGACTCTGCTGTTAATGCTATCTGCTATGGCGCCAAGCTCATGACTCCTGGGCTGCTCCGGTTTGAGAATGACAATGATGTTGGGGAAGAGGTTGTTCTCATGACCACCCCTTGCAACTGTCTATGGTTTGGTAATCAGAGGGACTCCCCATCAAGTTTCCTAGACAATGAGGCCTGTTTTGCTGTATCTGTAACTAGTTTCATGAAAAAGATAGTAGCAGCAACCTGTTGGAAGCTGGCCCTTAGTATTTTCCGTGTATGACAAGTGTAATAATGGGAACCATGTGTATTATGATGTACTAGTACAATTCTTCGTT
