# Supplementary material for: Transcriptome and proteome profiling revealed molecular mechanism of selenium responses in bread wheat (Triticum aestivum L.)
Source: BMC Plant Biol. 2021 Dec 9;21:584. doi: 10.1186/s12870-021-03368-w (PMC8656055; doi:10.1186/s12870-021-03368-w)
Supplement: Supplementary file 9 — Additional file 9: Supplementary material 9 Oligonucleotide primers used in qRT-PCR confirmation. [file 12870_2021_3368_MOESM9_ESM.docx]

Supplementary material 9: Oligonucleotide primers used in qRT-PCR confirmation.

| Gene ID | Annotation | Description | Primer Name | Primer Sequence (5’→3’) |
| --- | --- | --- | --- | --- |
| TraesCS4B02G325800  TraesCS4B02G325800  TraesCS6D02G154600  TraesCS6D02G154600  TraesCS5A02G325200  TraesCS5A02G325200  TraesCS6B02G051800  TraesCS6B02G051800  TraesCS6D02G177400  TraesCS6D02G177400  TraesCS3B02G334300  TraesCS3B02G334300  TraesCS3A02G457400  TraesCS3A02G457400  TraesCSU02G207700  TraesCSU02G207700  TraesCS5A02G238300  TraesCS5A02G238300  TraesCS7A02G130000  TraesCS7A02G130000  AB181991  AB181991 | *TaCAT*  *TaCAT*  *TaLOX*  *TaLOX*  *TaUDPGT*  *TaUDPGT*  *TaAT*  *TaAT*  *TaST*  *TaST*  *TaGST*  *TaGST*  *TaCULLIN*  *TaCULLIN*  *TaPC1*  *TaPC1*  *TaGDSL*  *TaGDSL*  *TaPT*  *TaPT*  *TaACTIN*  *TaACTIN* | catalase  catalase  lipoxygenase  lipoxygenase  UDP-glucuronosyltransferase  UDP-glucuronosyltransferase  acyl-tranferase  acyl-tranferase  sugar transporter  sugar transporter  glutathione S transferase  glutathione S transferase  cullin  cullin  peptidase C1  peptidase C1  Lipase GDSL  Lipase GDSL  prenyltransferase  prenyltransferase  actin  actin | TaCAT-Fq  TaCAT-Rq  TaLOX-Fq  TaLOX-Rq  TaUDPGT-Fq  TaUDPGT-Rq  TaAT-Fq  TaAT-Rq  TaST-Fq  TaST-Rq  TaGST-Fq  TaGST-Rq  TaCULLIN-Fq  TaCULLIN-Rq  TaPC1-Fq  TaPC1-Rq  TaGDSL-Fq  TaGDSL-Rq  TaPT-Fq  TaPT-Rq  TaACTIN-Fq  TaACTIN-Rq | CTCCACCGTGATCCACGAGC  GCCGTCGCGGATGAAGAAGA  CGAGGCGCTGAGGAAGAAGG  AGGGTAGGGGTGCTCCTTGG  CGCGTCTCCTTCGTCTCCAC  GAGGAAGGCGGAGAATGGGC  GATGGAAGGCCATGGACCCG  ACAGTTCCGCGGTTTACGCA  CGGCTTCGCTCGGTTCTGAT  GAGCGACATGGCCACGATGA  TACGTCTGGGAATCGCGTGC  GGCTCGCCCGAACATAGGAC  TCGATTCGTGCTCCGTTCCG  GCATTCAGCAAGGCAGCGAC  TTCATGCGCTCGTCGTCCTC  CCTCCCCAGCATCAGCAGTG  AGCAGCTGCGCTACTTCGAG  TAGCGGACGTAGTCCGGGAG  CCCCCTCTGGCCTGAGTTCT  AGCTGCTTCTTCGCCAGACC  CCAGCGGTCGAACAACTGGT  GGCCACGTAAGCGAGCTTCT |
